# A Near-Infrared Fluorescent Probe Based on a FRET Rhodamine Donor Linked to a Cyanine Acceptor for Sensitive Detection of Intracellular pH Alternations

**DOI:** 10.3390/molecules23102679

**Published:** 2018-10-18

**Authors:** Yibin Zhang, Jianheng Bi, Shuai Xia, Wafa Mazi, Shulin Wan, Logan Mikesell, Rudy L. Luck, Haiying Liu

**Affiliations:** 1 Department of Chemistry, Michigan Technological University, 1400 Townsend Drive, Houghton, MI 49931, USA; yibinz@mtu.edu (Y.Z.); jbi1@mtu.edu (J.B.); shuaix@mtu.edu (S.X.); wamazi@mtu.edu (W.M.); swan@mtu.edu (S.W.); ldmikese@mtu.edu (L.M.); 2 School of Chemistry and Chemical Engineering, Yangtze Normal University, Chongqing 408100, China

**Keywords:** near-infrared imaging, fluorescent probes, FRET, rhodamine, cyanine dye

## Abstract

A fluorescence resonance energy transfer (FRET)-based near-infrared fluorescent probe (**B^+^**) for double-checked sensitive detection of intracellular pH changes has been synthesized by binding a near-infrared rhodamine donor to a near-infrared cyanine acceptor through robust C-N bonds via a nucleophilic substitution reaction. To demonstrate the double-checked advantages of probe **B^+^**, a near-infrared probe (**A**) was also prepared by modification of a near-infrared rhodamine dye with ethylenediamine to produce a closed spirolactam residue. Under basic conditions, probe **B^+^** shows only weak fluorescence from the cyanine acceptor while probe **A** displays nonfluorescence due to retention of the closed spirolactam form of the rhodamine moiety. Upon decrease in solution pH level, probe **B^+^** exhibits a gradual fluorescence increase from rhodamine and cyanine constituents at 623 nm and 743 nm respectively, whereas probe **A** displays fluorescence increase at 623 nm on the rhodamine moiety as acidic conditions leads to the rupture of the probe spirolactam rings. Probes **A** and **B^+^** have successfully been used to monitor intracellular pH alternations and possess pK_a_ values of 5.15 and 7.80, respectively.

## 1. Introduction

Different cellular compartments regulate intracellular pH as precise control is essential for various cell functions such as vesicle trafficking, cellular metabolism, cellular signaling, cell membrane polarity, cell activation, proliferation growth, and apoptosis [1,2,3,4]. Intracellular pH values are quite different in different organelles [1,2,3,4]. Lysosomes function best under acidic pH conditions between 4.5 to 5.5 to break down a variety of biomolecules while mitochondria operate under slightly alkali pH conditions around 8.0 [4,5,6]. Various diseases such as neurodegenerative disease, cancer, and Alzheimer’s disease, are associated with significant deviations from normal functional intracellular pH values [1,2,3,4]. Therefore, monitoring intracellular pH levels is important in order to understand cellular functions.

**Scheme 1:**
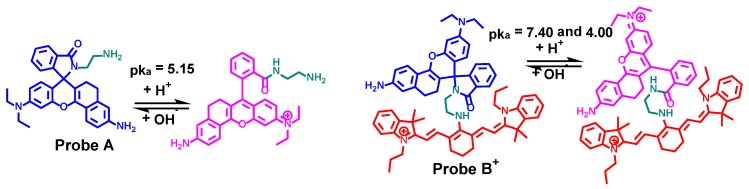
Drawings of the probes and their protonated versions.

Fluorescence imaging is frequently used for real-time pH monitoring in biological systems due to its rapid response time, high sensitivity, non-destructive nature, operational simplicity, and high-speed spatial capabilities [4]. Recently, near-infrared pH fluorescent probes were developed to take advantage of near-infrared imaging unique features such as minimum photobleaching, deep tissue penetration, suppressed photodamage to cells and tissues, and low biological luminescence background [5,6,7,8,9,10,11,12,13,14,15,16,17,18,19,20,21,22,23,24,25,26,27,28,29,30,31,32,33]. Most of these near-infrared fluorescent probes that measure pH levels are based on fluorescence changes in a single near-infrared wavelength [5,6,7,8,9,10,11,12,13,14,15,16,17,18,19,20,21,22,23,24,25,26,27,28,30,31,32,33]. We have developed a near-infrared fluorescent probe (**B^+^**), Scheme 1, with unique double-checked capability to accurately detect intracellular pH alternations by monitoring deep-red and near-infrared fluorescence changes at 623 nm and 780 nm. The probe’s double-checked feature was achieved by connecting a near-infrared rhodamine dye as a Forster resonance energy transfer (FRET) donor to a near-infrared cyanine dye as a FRET acceptor tethered via an ethylene-diamino linkage with robust C-N bonds. Probe **B^+^** possesses two pK_a_ values of 4.0 and 7.4 corresponding to spirolactam ring opening of the rhodamine donor, and protonation of the central nitrogen atom of the cyanine acceptor respectively under rhodamine donor excitation at 450 nm. This advantageous characteristic with two different pK_a_ values in one system enables us to determine pH changes in a broad range with both fluorescence increases of the rhodamine donor and cyanine acceptor. We also prepared a near-infrared fluorescent probe (**A**), Scheme 1, through modification of a near-infrared rhodamine dye with ethylenediamine. We demonstrate that there is slight overlap between the rhodamine donor emission and cyanine acceptor absorption for energy transfer from the rhodamine donor to the cyanine acceptor presumably through the short ethylenediamino linkage [34,35]. Probe **A** shows the expected fluorescence increase responses to pH variance under pH stimulus from 7.4 to 3.0. Probe **B^+^** exhibits weak fluorescence from the cyanine acceptor under basic pH conditions with retention of the closed spirolactam configuration. Gradual increase in acidity from pH 9.0 to 3.0 results in fluorescence increases with the rhodamine and the cyanine moieties under rhodamine donor excitation. This allows for accurate monitor of intracellular pH levels through two near-infrared channels. Probe **B^+^** shows excellent photostability, low cytotoxicity, good selectivity, and high sensitivity to pH near-infrared imaging feature. These conclusions are also confirmed by theoretical studies. 

## 2. Results

### 2.1. Synthesis of Fluorescent Probes

**Scheme 2:**
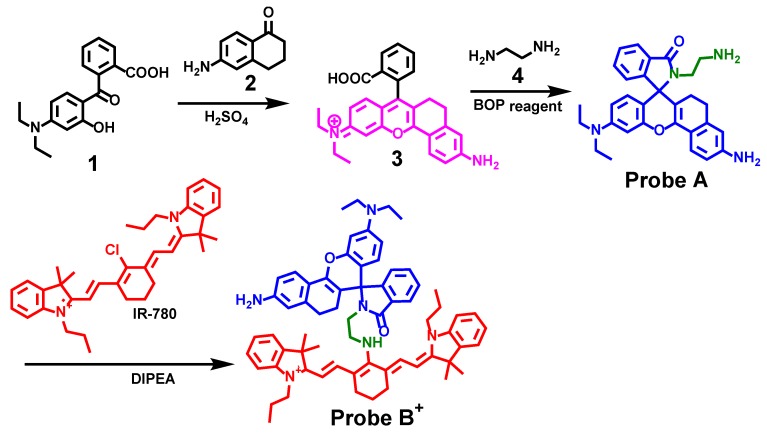
Synthetic route to probes **A** and **B^+^**.

In order to bind a near-infrared rhodamine donor to a cyanine acceptor which contains a reactive chloro site for chemical substitution, we prepared rhodamine dye (**3**) by a condensation reaction of 2-(4-(diethylamino)-2-hydroxybenzoyl) benzoic acid (**1**) and 6-amino-3,4-dihydro-1(2H)-naphthalenone (**2**) in concentrated sulfuric acid under reflux conditions. The rhodamine dye bearing a closed spirolactam ring with amine residue (probe **A**) was prepared by reacting rhodamine dye (**3**) with a large excess amount of 1,2-diaminoethane (**4**) in the presence of benzotriazol-1-yloxytris(dimethylamino)phosphonium hexafluorophosphate (BOP). Probe **B^+^** was synthesized through a nucleophilic substitution reaction of the central chloro group in a rigid chlorocyclohexenyl ring of cyanine dye (IR-780) with the dangling -NH_2_ group on probe **A** under basic conditions (Scheme 2). All intermediates and probes were characterized by ^1^H and ^13^C NMR and mass spectrometer as detailed in Supplementary Materials. 

### 2.2. Optical Responeses of Fluorescent Probes to pH Changes

**Figure 1:**
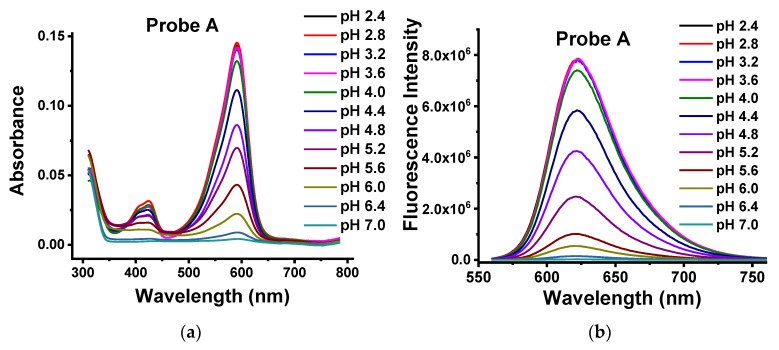
Absorption (**a**) and fluorescence spectra (**b**) of probe **A** in different pH buffers under excitation at 550 nm.

The optical pH-responsive properties of probes **A** and **B^+^** were investigated in different pH buffers containing 1% DMSO. Probe **A** is an intensity-based rhodamine dye with a spirolactam ring on/off switch, which can undergo ring opening/closing processes under pH stimulus. Probe **A** has an absorption at 300 nm but no emission with a closed spirolactam ring configuration under basic conditions (Figure 1). Upon gradual decrease in pH, both absorbances at 415 and 591 nm and fluorescence intensity at 623 nm increase due to acid-activated opening of the spirolactam structure, Figure 1. Therefore, probe **A** displays typical intensity-based fluorescence responses to pH changes, and possesses a pK_a_ value of 5.15 related to the spirolactam ring opening. Probe **A** shows a high fluorescence quantum yield of 31.1% under acidic conditions (pH 4.0). The molar absorbtivity is 2.88 × 10^4^ L·mol^−1^·cm^−1^ at 591 nm at pH 4.0.

**Figure 2:**
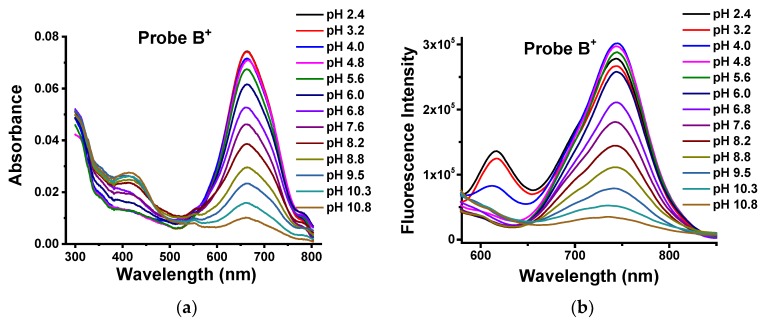
Absorption (**a**) and fluorescence spectra (**b**) of probe **B^+^** in different pH buffers under excitation at 450 nm.

In order to monitor pH changes and the assumed double-checked feature, probe **B^+^** was prepared by introducing a rhodamine donor to a cyanine acceptor through a very short ethylene spacer to achieve high efficiency of energy transfer from the donor to the acceptor. Probe **B^+^** shows a weak absorption peak at 664 nm and an extremely weak fluorescence peak at 743 nm, Figure 2. However, gradual pH decreases from 10.8 to 2.4 causes corresponding increases in the absorption peak, and results in a new absorption peak at 413 nm, and gradual increases of fluorescence peaks at 616 nm and 743 nm under excitation at 450 nm, Figure 2. Additionally, under cyanine acceptor excitation at 645 nm, the fluorescence intensity of the cyanine moiety also increases upon pH decrease, indicating that acidic pH results in protonation of the central amine atom of cyanine acceptor and increases in fluorescence of probe **B^+^** through the spirolactam ring opening.

**Figure 3:**
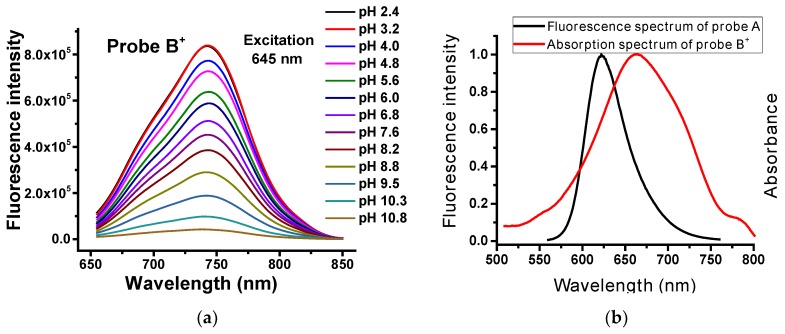
Fluorescence spectra of cyanine acceptor of probe **B^+^** in different pH buffers (**a**), fluorescence spectrum of probe A, and absorption spectrum of probe **B^+^** at pH 4.8 (**b**).

The introduction of a robust C-N bond to the cyanine moiety results in a blue shift of absorption of the cyanine acceptor with absorption around 650 nm which allows for efficient FRET processes as the absorption of the cyanine acceptor overlaps with the emission from the rhodamine donor [34,35]. Since there is significant overlap between the fluorescence of the rhodamine donor (probe **A**) and absorption of the cyanine acceptor (probe **B^+^**) (Figure 3, right), excitation at 450 nm allows for an effective FRET process from the rhodamine donor to the cyanine acceptor, resulting in both fluorescence increases of the rhodamine donor and the cyanine acceptor with decreases in pH (Figure 2). It is also noteworthy that the intensity of the fluorescence from the cyanine acceptor has a much more significant increase than the intensity of the fluorescence from the rhodamine donor presumably due to the FRET process for probe **B^+^**, which in the case of the rhodamine moiety in isolated probe **A** (free of the cyanine moiety) was quite substantive. Probe **B^+^** shows a quantum yield of 12.4% (pH 4.0) under 450 nm excitation using human dye as standard. The molar absorptivity is 1.50 × 10^4^ L·mol^−1^·cm^−1^ at 663 nm at pH 4.0. The rhodamine donor of probe **B^+^** has a pK_a_ value of 4.0 due to spirolactam ring opening of the rhodamine donor while the probe cyanine acceptor has a higher pK_a_ value of 7.4 arising from protonation of the central nitrogen atom of the cyanine acceptor under rhodamine donor excitation at 450 nm. This nice feature with two different pK_a_ values in one system enables to determine pH changes in a broad range with both fluorescence increases of the rhodamine donor and cyanine acceptor. The efficiency of FRET from the rhodamine donor to the cyanine acceptor was calculated to be 23.5% in pH 3.2 buffer since the FRET efficiency depends on not only the distance, but also the overlap between donor’s emission and acceptor’s absorption spectra and orientation.

**Figure 4:**
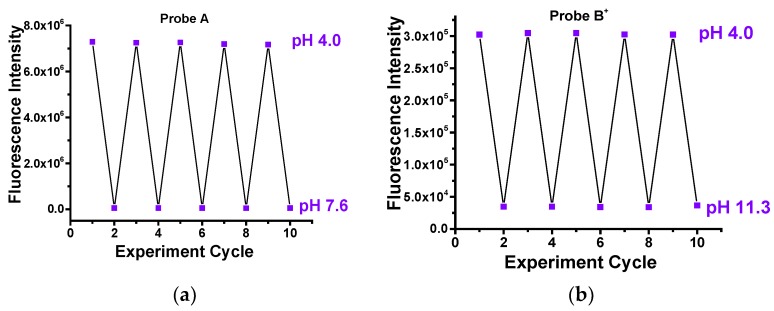
Fluorescence intensities of probes **A** (**a**) and **B^+^** (**b**) in different pH conditions under 450 nm excitation for probe **B^+^**, and 550 nm excitation for probe **A**.

**Figure 5:**
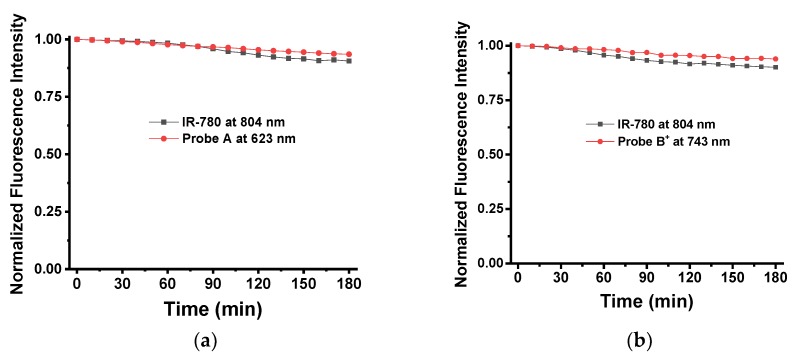
Fluorescence intensity of probes **A** (**a**) and **B^+^** (**b**) under continual excitation of 450 nm for probe **B^+^** and 550 nm for probe **A**.

Probe **A** reversibly responds to pH changes between 4.0 and 7.6, Figure 4. Probe **B^+^** under 450 nm excitation also shows excellent reversible fluorescence responses at 743 nm to pH changes between 4.0 and 10.8. The results indicate that probes **A** and **B^+^** are stable and demonstrate reversibility to changes in pH, Figure 4, as compared to the IR-780 dye, Figure 5.

### 2.3. Selectivity the Probes to pH over Cations, Anions, and Amino Acids

**Figure 6:**
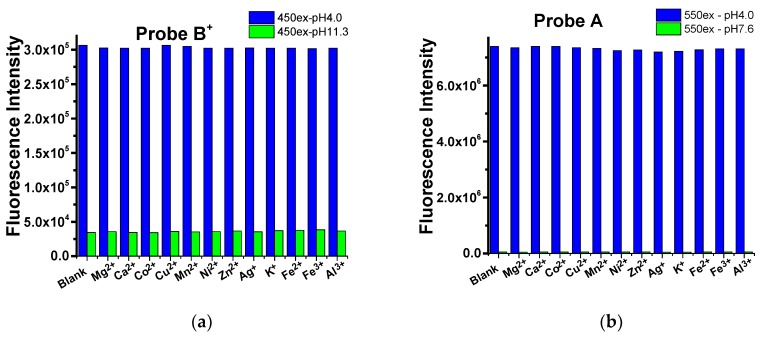
Fluorescence intensity of probes **A** and **B^+^** in the absence and presence of 50 µM different metal ions at pH 4.0 and 11.3 under 450 nm excitation for probe **B^+^** (**a**) and under 550 nm excitation for probe **A** (**b**).

**Figure 7:**
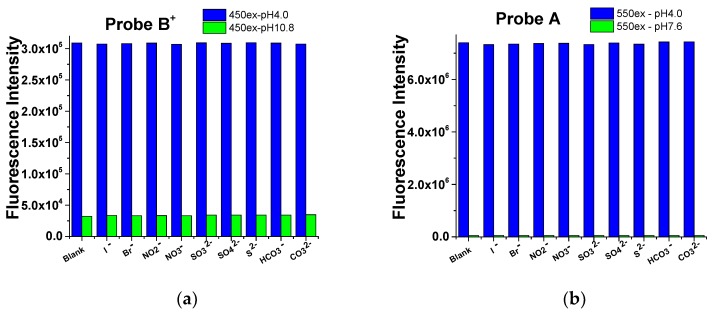
Fluorescence intensity of probes **A** and **B^+^** in the absence and presence of 50 µM different anions at pH 4.0 and 11.3 under excitation of 450 nm for probe **B^+^** (**a**) and under 550 nm excitation for probe **A** (**b**).

**Figure 8:**
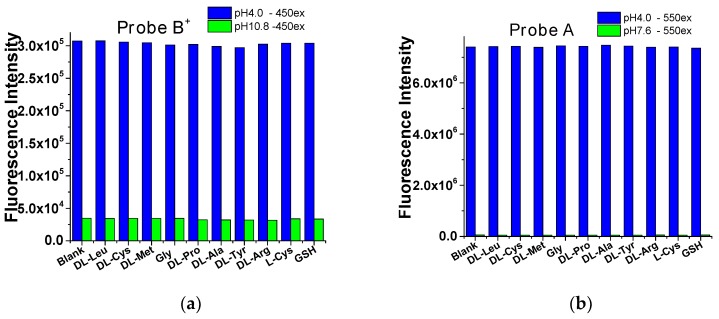
Fluorescence intensity of probes **A** and **B^+^** in the absence and presence of 50 µM different amino acid and biothiols at pH 4.0 and 11.3 under excitation of 450 nm for probe **B^+^** (**a**) and under 550 nm excitation for probe **A** (**b**).

We studied the responsiveness of probes **A** and **B^+^** to pH to metal ions. Essential metal ions including K^+^, Cu^2+^, Mg^2+^, Mn^2+^, Ni^2+^, Ag^+^, Fe^3+^, Fe^2+^, Al^3+^, and Co^2+^ ions were added to solutions of probe **A** or **B^+^** at pH 4.0, 7.6, or 11.3, and, only insignificant changes of fluorescence intensity were detected, Figure 6. Further, common anions such as I^−^, Br^−^, NO^2−^, NO^3−^, SO_4_^2−^, SO_3_^2−^, S^2−^, HCO_3_^−^, and CO_3_^2−^ ions also display little influence on the fluorescence intensity of probe **A** or **B^+^** under pH 4.0, 7.6 or 11.3, Figure 7. Finally, amino acids and nucleophilic biothiols such as leucine, methionine, alanine, proline, arginine, threonine, glycine, cysteine, and glutathione have small effects on fluorescence responses at pH levels of 4.0, 7.6 or 11.3, Figure 8.

### 2.4. Theoretical Results

**Figure 9:**
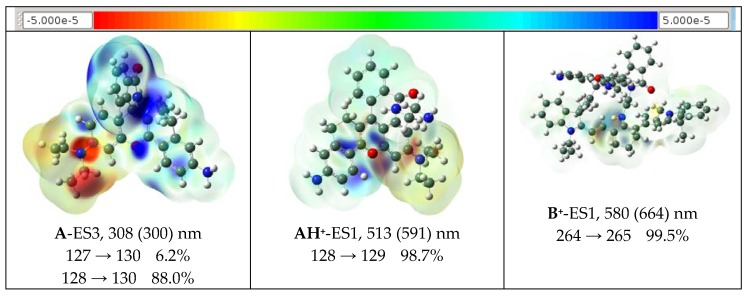

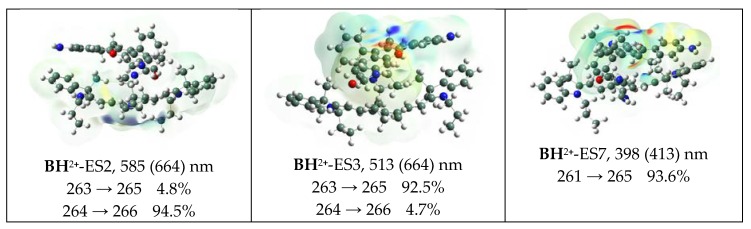
Current density difference illustrations as iso-surfaces of the probes as indicated for the excited states (ES) and the calculated and (experimental) wavelength. The composition of that specific ES together with percentage contribution is also indicated. Drawings of the numbered linear combination of atomic orbitals (LCAOs) are available in Supporting Information. Red areas indicate values for the different density of −5.000 × 10^−5^ and blue are for 5.000 × 10^−5^, see scale on top of illustration.

The drawings and data in Figure 9 summarize pertinent results from theoretical calculations based on density functional theory DFT-APFD [36], at the 6-311+G(2d, p) [37,38,39] level implemented using Gaussian 16 [40]. We find reasonable agreement with the experimental and calculated transitions for the probes as listed in Figure 9. The results suggest that the transition for probe **A** consists of electron movement from the diethylamino moiety onto the spirolactam section of the molecule. With probe AH^+^ which does not contain the spirolactam ring, more even π-delocalization pertains, judging by the lack of intense blue. The transition also emanates from the diethylamino moiety, namely ES 1, and occurs at 513 nm. Probe B^+^ which contains the spirolactam ring revealed a ES transition at 580 nm with 99.5% percent localization on the cyanine moiety. Protonation of probe B^+^ to produce probe BH^2+^ results in three ES transitions of suitable oscillator strength to be considered, see Table S8. The transition ES 2 at 585 nm has a 94.5% basis on π to π* orbitals localized on the cyanine moiety with the rest (i.e., 4.8%) from π to π* orbitals localized on the rhodamine moiety. This situation is reversed with the ES 3 transition with a 92.5% composition from the rhodamine sections and 4.7% from the cyanine. This is clearly observable in the sections of probe BH^2+^ that are colored light blue in Figure 9. Clearly in an experimentally obtained absorption spectrum, these contributions would overlap and result in a broad transition at 550 nm (expt 664 nm), see Figure S28. A higher energy transition consisting of a lower energy π-delocalize (i.e., HOMO-3) orbital to the LUMO localized on the rhodamine section occurring at 398 nm (expt 413 nm) is also calculated. The results of this calculation do not reveal any transitions from the rhodamine to the cyanine moieties in probe BH^2+^. This adds credence to the likelihood of FRET transfer as an explanation of the aforementioned absorption and fluorescence data.

### 2.5. Cellular Fluorescence Imaging

**Figure 10:**
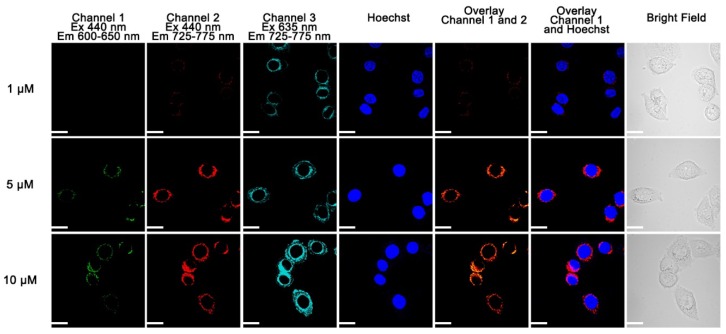
Live-cell fluorescence images of HeLa cells incubated with probe **B^+^** for 2 h and then with Hoechst stain for 1 h with scale bars at 20 μm. The excitation of Hoechst is 405 nm and the fluorescence collect window is from 425 to 475 nm.

**Figure 11:**
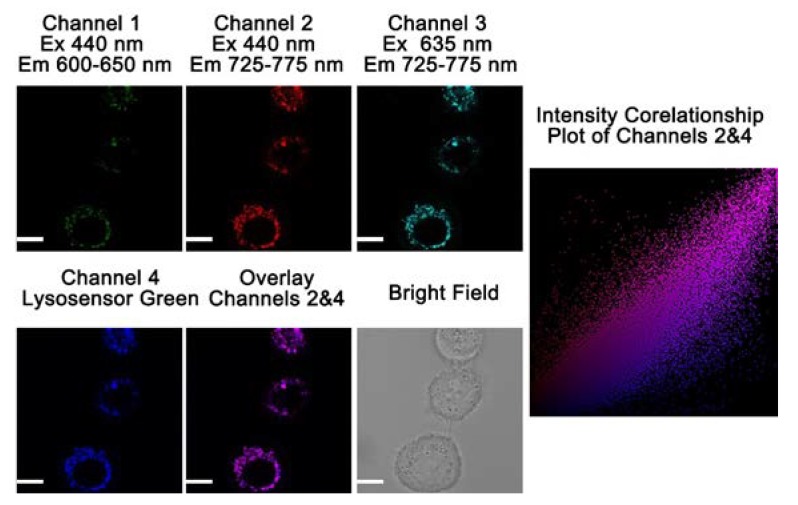
Live-cell fluorescence images of HeLa cells incubated with probe **B^+^** for 2 h and then with Lysosensor Green for 1 h with scale bars at 20 μm. The excitation of Lysosensor green is 405 nm and the fluorescence collect window is from 500 to 550 nm.

A confocal fluorescence microscope was used to conduct cellular fluorescence imaging of HeLa incubated with probe **B^+^**. The cellular fluorescence intensity of probe **B^+^** increases with probe concentration from 1 μM to 10 μM as deep-red fluorescence from 600 nm to 650 nm, and near-infrared fluorescence from 725 nm to 775 nm can be clearly observed under rhodamine excitation at 440 nm with 10-μM concentration level of probe **B^+^**, Figure 10. Strong cellular near-infrared fluorescence can be observed under cyanine acceptor excitation at 635 nm with 1 μM concentration of probe **B^+^** due to high fluorescence quantum yield under acidic pH condition. These results demonstrate that probe **B^+^** effectively stain the cells. We also conducted a colocalizaton imaging experiment of probe B with Lysosensor Green in HeLa, and obtained a high Pearson collocalization coefficient of 0.93 between probe B and Lysosensor Green, indicating that probe B is located in lysosomes in HeLa cells, Figure 11.

**Figure 12:**
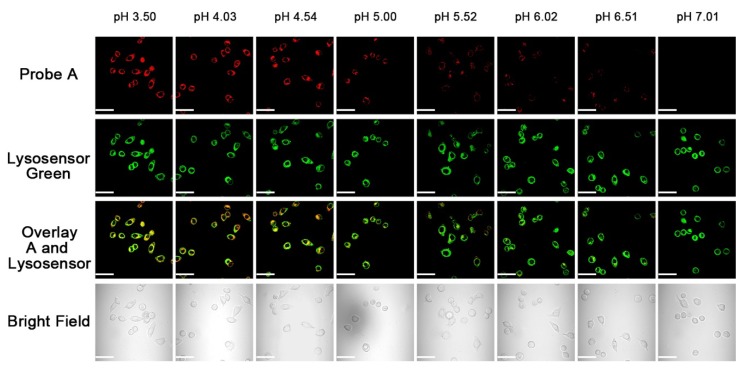
Confocal fluorescence images of HeLa cells incubated with probe **A** and Lysosensor Green in different pH buffers containing 10 µM nigericine with scale bars at 50 μm. The excitation of Lysosensor green is 405 nm and the fluorescence collect window is from 500 to 550 nm.

**Figure 13:**
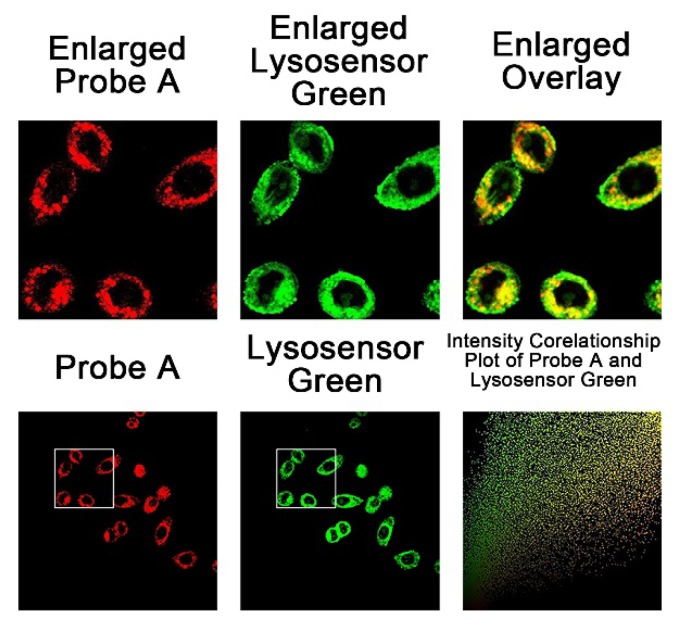
Enlarged confocal fluorescence images of HeLa cells incubated with probe **A** and Lysosensor Green in pH 3.50 buffer containing 10 µM nigericine from Figure 12.

Probe **A** as a weak base bearing an amine residue, should function as a lysosome-targeting imaging agent to selectively stain lysosomes in live cells. In order to test this hypothesis, we conducted colocalization experiments by using commercial Lysosensor Green to determine the intracellular location of probe **A**. Intracelluar pH values were adjusted by incubating HeLa cells in different pH buffers containing 10 µM nigericine, H^+^ ionphore, which is employed to promote equilibration between intracellular and extracellular pH values [7,9,29,41,42]. Probe **A** responds sensitively to intracellular pH decreases from 7.01 to 3.50 with gradual fluorescence enhancement due to acid-activated spirolactam ring opening with significantly enhanced π-conjugation, Figure 12. The respective Pearson’s colocalization coefficents between Lysosensor Green and probe **A** were higher than 0.86 under acidic pH 3.50 or 4.03, Figure 13, indicating that probe **A** selectively stains lysosomes in live cells with pH-sensitive responses while Lysosensor Green is insensitive to pH changes, see Figure 12. The plot of the average fluorescence intensities of probe **A** in HeLa cells versus pH gave a pK_a_ value of 5.20. 

**Figure 14:**
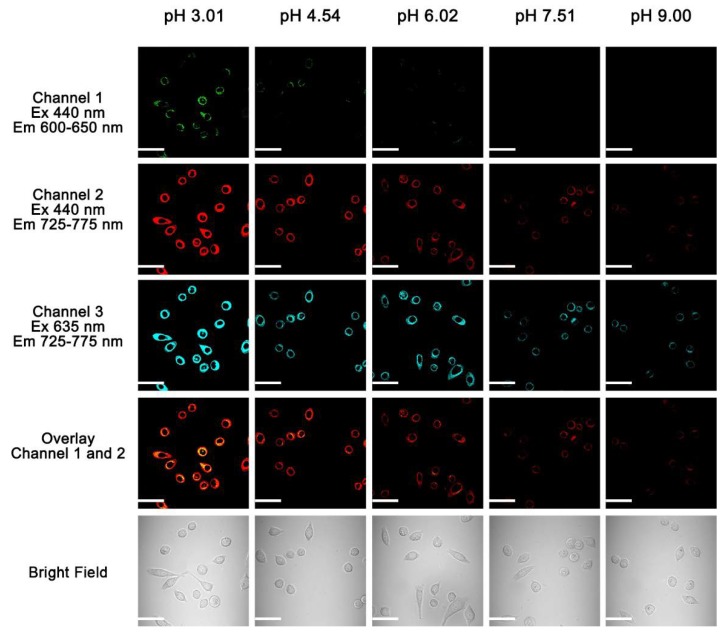
Confocal fluorescence images of HeLa cells incubated with probe **B^+^** in different pH buffers containing 10 µM nigericine with scare bars at 50 μm.

We also investigated whether probe **B^+^** could be used to detect intracellular pH changes with double-checked feature by incubating HeLa cells with probe **B^+^** in different pH buffers containing 10 µM nigericine, Figure 14. Gradual decreases of intracellular pH values from 9.00 to 3.01 cause increases of deep-red fluorescence in channel 1 and near-infrared fluorescence in channel 2 under rhodamine donor excitation at 440 nm. In addition, intracellular near-infrared fluorescence in channel 3 under cyanine excitation at 635 nm also increases with the same gradual pH decreases, demonstrating that probe **B^+^** can sensitively monitor intracellular pH changes.

## 3. Discussion

Fluorescence imaging with molecular fluorescent probes serves as an important tool in biological and medical research. Near-infrared imaging provides many advantages with prolonged fluorescence with less photobleaching problems, deep tissue penetration, and low interference from biological fluorescence backgrounds. Fluorescent probe **A** was measured to have a high pK_a_ value of 5.15 corresponding to spirolactam ring opening although only 1,2-diaminoethylene was used to modify the deep-red rhodamine dye. Probe **A** effectively targets lysosomes in live cells and can detect pH changes in lysosomes with a very suitable pK_a_ value since lysosomes are membrane-encased organelles with optimal pH from 4.5 to 5.0 for the enzyme activity in hydrolysis to degrade biological species, Figure 12. Compared with probe **A**, a commercial lysosensor is insensitive to pH changes although it can specifically target lysosomes in live cells. We further employed deep-red rhodamine as a FRET donor and near-infrared cyanine as a FRET acceptor to detect intracellular pH changes in live cells with a double-checked feature. Since the emission of the rhodamine donor has significant overlap with the absorption of the cyanine acceptor, efficient energy transfer from the rhodamine donor to the cyanine acceptor occurs through a very short ethylene spacer under acidic pH conditions. Probe **B^+^** containing the cyanine and rhodamine moieties, shows corresponding fluorescence increases with pH decreases to achieve the double-checked capability. Finally, we have developed ratiometric near-infrared fluorescent probes by introducing a spirolactam on/off switch to a cyanine acceptor which when activated results in a deep-red rhodamine donor connected to a cyanine acceptor through an ester bond instead of a spirolactam ring with an amide bond. 

## 4. Materials and Methods 

### 4.1. Synthesis of Probe A

After compound 3 [43] (439 mg, 1 mmol), ethylenediamine (180 mg, 3 mmol), BOP reagent (530 mg, 1.2 mmol) and trimethylamine (1 mL) were added to dry dichloromethane (10 mL), the mixture was stirred at room temperature for 16 h. The mixture was diluted with dichloromethane, washed with water and brine, dried with anhydrous Na_2_SO_4_, and filtered, and the filtrant concentrated under reduced pressure. The resulting residue was purified by using flash column chromatography through gradient elution with methanol ratio to dichloromethane from 5% to 10%. Probe **A** was obtained as blue solid. ^1^HNMR (300 MHz, CDCl_3_) δ: 7.78 (d, *J* = 7.2 Hz, 1H), 7.59 (d, *J* = 8.2 Hz, 1H), 7.38 (p, *J* = 7.2 Hz, 2H), 7.09 (d, *J* = 7.1 Hz, 1H), 6.55 (d, *J* = 8.2 Hz, 1H), 6.45–6.28 (m, 3H), 6.23 (d, *J* = 8.7 Hz, 1H), 4.02 (s, 4H), 3.43–3.65 (m, 1H), 3.25–3.30 (m, 5H), 2.78–2.56 (m, 2H), 2.46–2.56 (m, 2H), 1.73–1.41 (m, 2H), 1.11 (t, *J* = 6.9 Hz, 6H); 13CNMR (75 MHz, CDCl3) δ: 169.61, 152.90, 151.57, 148.86, 147.50, 138.31, 132.71, 131.45, 128.55, 128.49, 123.71, 123.63, 123.03, 120.14, 114.21, 112.68, 108.95, 104.77, 100.44, 98.06, 67.20, 44.57, 42.05, 41.18, 28.52, 21.40, 12.93. LCMS (ESI): calculated for C_30_H_32_N_4_O_2_ [M]^+^ 481.2, found 481.5.

### 4.2. Synthesis of Probe B^+^

Probe **A** (240 mg, 0.5 mmol), cyanine dye (IR-780) (400 mg, 0.6 mmol), *N*,*N*-diisopropylethylamine (DIPEA) (129 mg, 1 mmol) were added to acetonitrile (10 mL). The mixture was refluxed for 2 h and the reaction solution concentrated under reduced pressure. The resulting residue was purified by using flash column chromatography through gradient elution with methanol ratio to dichloromethane from 0% to 5%. Probe **B^+^** was obtained as blue solid. 1HNMR (300 MHz, CDCl_3_) δ: 9.28 (s, 2H), 7.83 (d, *J* = 7.5 Hz, 2H), 7.65–7.47 (m, 5H), 7.18–7.31 (m, 5H), 7.06 (t, *J* = 7.5 Hz, 2H), 6.87 (d, *J* = 7.8 Hz, 2H), 6.60 (d, *J* = 8.2 Hz, 1H), 6.54 (s, 1H), 6.44–6.24 (m, 2H), 5.60 (d, *J* = 12.7 Hz, 2H), 3.95–4.19 (m, 4H), 3.84–3.69 (m, 4H), 3.65–3.51 (m, 2H), 3.50–3.40 (m, 2H), 3.34 (t, *J* = 7.4 Hz, 4H), 2.65–2.52 (m, 2H), 2.52–2.39 (m, 4H), 1.84–1.71 (m, 6H), 1.54 (d, *J* = 10.4 Hz, 10H), 1.14 (t, *J* = 7.0 Hz, 6H), 0.98 (t, *J* = 7.4 Hz, 6H); 13CNMR (75 MHz, CDCl3) δ: 170.89, 167.51, 153.27, 149.07, 148.07, 143.25, 140.17, 137.44, 133.54, 129.01, 128.38, 128.24, 124.11, 123.86, 123.01, 122.10, 119.26, 114.15, 112.83, 109.09, 108.97, 98.26, 94.64, 68.13, 53.02, 47.84, 45.14, 44.71, 41.75, 28.92, 28.82, 26.46, 21.85, 21.43, 20.42, 13.00, 12.11. LCMS (ESI): calculated for C_66_H_75_N_6_O_2_ [M]^+^ 983.5, found 983.5.

### 4.3. Theoretical Calculations

Chemdraw structures of probes A, AH^+^, B^+^ and BH^2+^ were optimized initially with the MM2 functionality in Chem3D and then further with Avogadro [44,45]. Calculations were then conducted using density functional theory (DFT) with spherical atom dispersion terms, namely APFD [36], with all electron basis sets at the 6-311+G(2d, p) [37,38,39] level implemented using the Gaussian16 suite of programs [40] for the full geometry optimization and frequency calculations of the probes. Imaginary frequencies were not obtained in any of the frequency calculations. The first six excited states (more if required) were assessed on the basis of TD-DFT optimizations [46] in a Polarizable Continuum Model (PCM) of water [47]. Results were interpreted using GaussView [48] for all data and figures. The diagrams and listings of atomic positions from the calculations, calculated IR and NMR spectra in some cases, listings of excited states with drawings of referenced LCAOs are supplied as supporting information.

### 4.4. Cell Culture and Cell Imaging Procedures

HeLa cells were cultured in modified Eagle’s medium (DMEM, Gibco, Carlsbad, CA, USA) containing 10% fetal bovine serum (FBS, fisher Scientific, Hampton, NH, USA) at 37 °C in humidified air with 5% CO_2_. HeLa cells were subcultured with 0.25% trypsin (*w*/*v*) every 2–3 day reached at 80% confluence.

For confocal live cell imaging, HeLa cells were seeded into the 35 nm glass-bottom culture dishes (MatTek, Ashland, MA, USA) and allowed 1 day to reach 50% confluence. After 24 h of incubation, the cell culture medium was replaced by freshly prepared FBS-free medium with 1, 5, and 10 μM of probe **B** for 1 h 37 °C under 5% CO_2_ followed by using PBS buffer to rinse three times. For the live cell fluorescence imaging at different pH, the HeLa cells were treated with 10 μM probe **A** or **B** 37 °C under 5% CO_2_ for 1 h. The cells were rinsed with PBS buffer twice before they were treated with 10 μM nigericin in citric buffer with pH values at 3.50, 4.03, 4.54, 5.00, 5.52, 6.02, 6.51, 7.01 for probe **A** and 3.01, 4.54, 6.02, 7.51, 9.00 for probe **B**, respectively, for 30 min to equilibrate the intracellular and extracellular pH for 30 min. The cells were rinsed with PBS buffer twice again before imaging.

## 5. Conclusions

A new FRET-based near-infrared fluorescent probe (**B^+^**) for pH sensing with double-checked feature was successfully prepared by conjugating a near-infrared rhodamine donor to a cyanine acceptor via a robust C-N bond connection with a short ethylene tethered spacer. Probe **A** was also prepared by introducing 1,2-diaminoethylene to rhodamine forming a closed spirolactam ring structure. Probe **B^+^** responds to pH decreases with fluorescence increases of both deep-red fluorescence of rhodamine donors and near-infrared fluorescence of the cyanine acceptor under rhodamine donor excitation at 450 nm.

## Supplementary Materials

The following are available online.
