# Supplementary material for: A Near-Infrared Fluorescent Probe Based on a FRET Rhodamine Donor Linked to a Cyanine Acceptor for Sensitive Detection of Intracellular pH Alternations
Source: Molecules. 2018 Oct 18;23(10):2679. doi: 10.3390/molecules23102679 (PMC6222743; doi:10.3390/molecules23102679)
Supplement: Supplementary file 1 [file molecules-23-02679-s001.pdf]

molecules

Supplementary Material

A Near-infrared Fluorescent Probe Based on a FRET Rhodamine Donor linked to a Cyanine Acceptor for Sensitive Detection of Intracellular pH Alternations

Yibin Zhang [1,2], Jianheng Bi [1], Shuai Xia [1], Wafa Mazi [1], Shulin Wan [1], Logan Mikesell [1], Rudy L. Luck [1,*] and Haiying Liu [1,*]

[1] Department of Chemistry, Michigan Technological University, 1400 Townsend Drive, Houghton, MI 49931, USA; yibinz@mtu.edu (Y.Z.); jbi1@mtu.edu (J.B.); shuaix@mtu.edu, wamazi@mtu.edu (S.X.); swan@mtu.edu (W.M.); ldmikese@mtu.edu (L.M.)
[2] School of Chemistry and Chemical Engineering, Yangtze Normal University, Chongqing 408100, China
* Correspondence: rluck@mtu.edu (R.L.L.); hyliu@mtu.edu (H.L.); Tel.: +1-906-487-3451 (H.L.)

Contents

Figure S1. Drawing of probe A with atoms represented as spheres of arbitrary size (H-white, C-grey, N-blue and O-red) using the GaussView [1] program....... 3

Table S1. Atomic coordinates for probe A....... 3

.... 4

Figure S2. LCAO for orbitals 127, 128 and 130 in probe A....... 4

Figure S3. Calculated UV-Vis spectrum for probe A....... 5

Table S2. Excitation Energies and Oscillator Strengths for A....... 5

Figure S4. Calculated FTIR spectrum of probe A in water....... 6

Figure S5. Calculated FTIR spectrum of probe A in gas phase without solution....... 6

Figure S6. The solid state FTIR spectrum of probe A....... 6

Figure S7. Tentative assignments for the proton NMR spectrum of probe A. This was based in part on the calculated spectrum shown below....... 7

Figure S8. Calculated proton NMR spectrum of probe A....... 7

Figure S9. Tentative assignments for the 13C NMR spectrum of probe A....... 8

Figure S10. Calculated ^{13}C NMR spectrum for probe A....... 8

Figure S11. Drawing of probe AH^+ with atoms represented as spheres of arbitrary size (H-white, C-grey, N-blue and O-red) using the GaussView[1] program....... 9

Table S3. Atomic coordinates for probe AH^+....... 9

Figure S12. LCAO for orbitals 127, 128 and 129 in probe AH^+....... 11

Figure S13. Calculated UV-Vis spectrum for probe AH^+....... 11

Table S4. Excitation Energies and Oscillator Strengths for AH^+....... 12

Figure S14. Calculated FTIR spectrum of probe AH^+ in water....... 12

Figure S15. Drawing of probe B^+ with atoms represented as spheres of arbitrary size (H-white, C-grey, N-blue and O-red) using the GaussView[1] program....... 13

Figure S16. Drawing of probe B^+ with atoms represented as spheres of arbitrary size (C-grey, N-blue and O-red) using the GaussView[1] program. H-atoms are not depicted for clarity 13

Table S5. Atomic coordinates for probe B^+. 13

Figure S17. LCAO for orbitals 264 and 265 in probe B^+. 16

Figure S18. Calculated UV-Vis spectrum for probe B^+. This represents a HOMO-LUMO transition. 17

Table S6. Excitation Energies and Oscillator Strengths for B^+. 17

Figure S19. Calculated FTIR spectrum of probe B^+ in water. 18

Figure S20. Current density difference plot for probe B^+ obtained by subtracting the SCF (ground state) density from the CI (excited state) density using the Cubegen program in GaussView. 18

Figure S21. Tentative assignments for the ^1H NMR spectrum of probe B^+. This was based in part on the calculated spectrum shown below 19

Figure S22. Calculated ^1H NMR spectrum of probe B^+. 19

Figure S23. Partial assignments of the ^{13}C NMR spectrum of probe B^+. 20

Figure S24. Calculated ^{13}C NMR spectrum of probe B^+. 20

Figure S25. Drawing of probe BH^{2+} with atoms represented as spheres of arbitrary size (H-white, C-grey, N-blue and O-red) using the GaussView [1] program. 21

Figure S26. Drawing of probe BH^{2+} with atoms represented as spheres of arbitrary size (C-grey, N-blue and O-red) using the GaussView [1] program. H-atoms are not depicted for clarity 21

Table S7. Atomic coordinates for probe BH^{2+}. 22

Figure S27. LCAO for orbitals 261, 263-266 in probe BH^{2+}. 24

Figure S28. Calculated UV-Vis spectrum for probe BH^{2+}. 24

Table S8. Excitation Energies and Oscillator Strengths for BH^{2+}. 24

Figure S29. Calculated FTIR spectrum of probe BH^{2+} in water. 2

Synthetic route of near-infrared Probes A and B 3

^1H NMR and ^{13}C NMR spectra of probes A and B 4

Figure S30. ^1H NMR spectrum of probe **A** in $CDCl_3$ solution 4

Figure S32. ^1H NMR spectrum of Probe B in $CDCl_3$ solution 5

Figure S33. ^{13}C NMR spectrum of probe B in $CDCl_3$ solution 5

Figure S34. The fluorescence ratio of cyanine acceptor to rhodamine donor of Probe B versus different pH values under rhodamine donor excitation of 450 nm 5

References. 7

General information on the theoretical calculations:

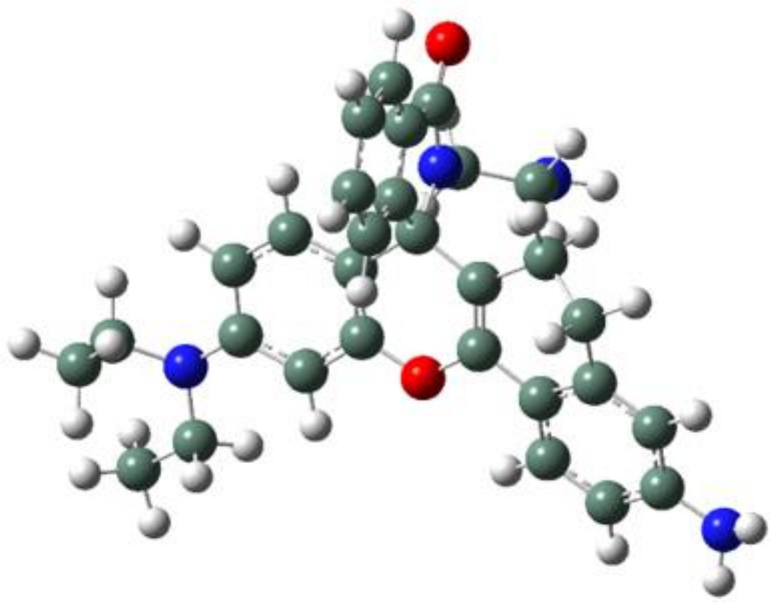

Figure S1. Drawing of probe A with atoms represented as spheres of arbitrary size (H-white, C-grey, N-blue and O-red) using the GaussView [1] program.

Table S1. Atomic coordinates for probe A.

Row	Symbol	X	Y	Z	Row	Symbol	X	Y	Z
1	C	-0.26462	1.312605	0.052673	24	N	-0.41938	2.050901	1.3298
2	C	1.024642	0.538962	0.037971	25	C	-0.49101	3.399696	1.185293
3	C	1.04219	-0.84444	-0.03685	26	C	-0.40591	3.665164	-0.26843
4	O	-0.09482	-1.58946	-0.16105	27	C	-0.26088	2.460175	-0.93131
5	C	-1.28965	-0.9346	-0.24949	28	C	-0.44844	4.871317	-0.95003
6	C	-1.43221	0.397719	-0.17831	29	C	-0.34176	4.83537	-2.33514
7	C	-2.42515	-1.83827	-0.39312	30	C	-0.19889	3.617676	-3.00619
8	C	-3.6596	-1.30454	-0.79661	31	C	-0.15923	2.41358	-2.30943
9	C	-3.72371	0.156096	-1.1445	32	C	-0.30628	1.409205	2.615486
10	C	-2.81595	0.982919	-0.24317	33	O	-0.59095	4.218615	2.094266
11	C	2.261907	1.175251	0.145127	34	C	-1.62045	0.851352	3.147005
12	C	3.448307	0.479202	0.182188	35	N	-1.40272	0.305511	4.484296
13	C	3.462696	-0.93623	0.122288	36	N	-5.79638	-4.30902	-0.71467
14	C	2.223275	-1.57665	-0.00938	37	H	-3.394	0.276058	-2.18605
15	C	-2.3314	-3.2057	-0.13449	38	H	-4.75406	0.517024	-1.09566
16	C	-3.4351	-4.03174	-0.25893	39	H	-2.76636	2.011681	-0.61005
17	C	-4.67129	-3.50558	-0.65133	40	H	-3.24563	1.040426	0.766031
18	C	-4.76016	-2.13262	-0.91952	41	H	2.285825	2.259662	0.205534
19	C	5.852513	-3.84281	0.322585	42	H	4.37051	1.038034	0.26115
20	C	4.571424	-3.09221	0.003429	43	H	2.135679	-2.65013	-0.10012
21	N	4.642174	-1.64976	0.213715	44	H	-1.38078	-3.62699	0.172788
22	C	5.889651	-0.98476	-0.13759	45	H	-3.34564	-5.09324	-0.04756
23	C	6.064038	-0.76253	-1.63559	46	H	-5.71194	-1.71128	-1.23298
					47	H	5.651786	-4.91508	0.264814

48	H	6.658576	-3.62477	-0.38105
49	H	6.204983	-3.62197	1.333634
50	H	3.786491	-3.47722	0.658949
51	H	4.25717	-3.31836	-1.02757
52	H	5.950848	-0.0397	0.402876
53	H	6.709386	-1.58668	0.252516
54	H	7.012685	-0.26028	-1.84261
55	H	6.061272	-1.71358	-2.17519
56	H	5.256766	-0.14401	-2.03632
57	H	-0.5624	5.807988	-0.4145
58	H	-0.37048	5.758506	-2.90437
59	H	-0.11813	3.613359	-4.08841
60	H	-0.0519	1.467566	-2.8305
61	H	0.434196	0.606654	2.525747
62	H	0.085512	2.146335	3.319904
63	H	-2.34823	1.664842	3.218012
64	H	-2.01754	0.115805	2.435762
65	H	-2.28515	0.000472	4.881165
66	H	-0.82447	-0.52775	4.426154
67	H	-6.5375	-3.97696	-1.3143
68	H	-5.62085	-5.29472	-0.84332

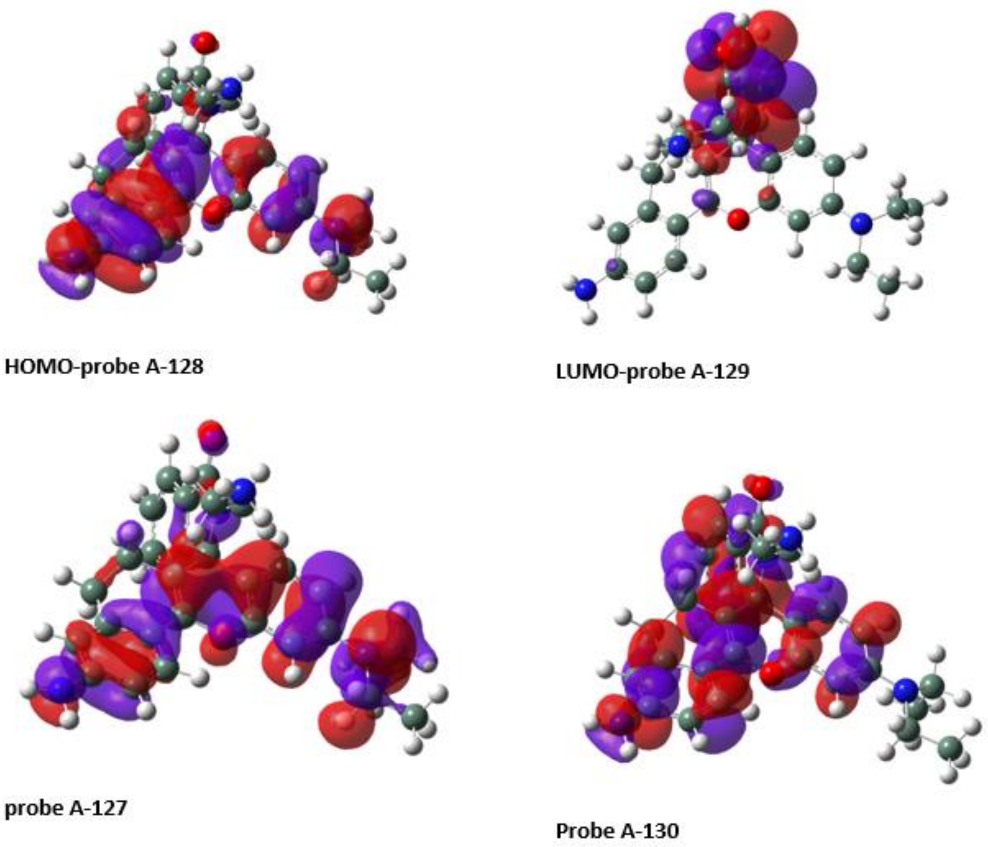

Figure S2. LCAO orbitals 127, 128 and 130 in probe A.

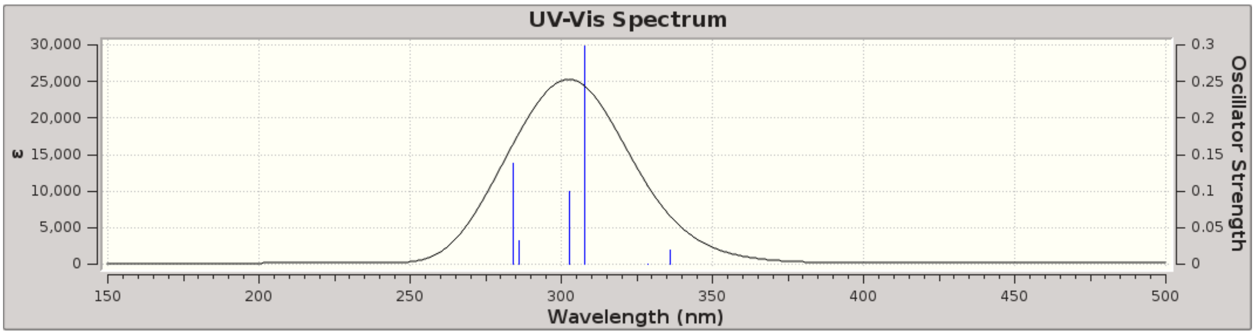

Figure S3. Calculated UV-Vis spectrum for probe A.

Table S2. Excitation Energies and Oscillator Strengths for A.

Excited State 1: Singlet-A 3.6890 eV 336.09 nm f=0.0194 <S**2>=0.000
127 -> 129 -0.21306
128 -> 129 0.66498
This state for optimization and/or second-order correction.
Total Energy, E(TD-HF/TD-DFT) = -1531.04901172
Copying the excited state density for this state as the 1-particle RhoCI density.

Excited State 2: Singlet-A 3.7732 eV 328.60 nm f=0.0005 <S**2>=0.000
127 -> 129 0.66239
128 -> 129 0.22173

Excited State 3: Singlet-A 4.0279 eV 307.81 nm f=0.2988 <S**2>=0.000
127 -> 130 0.17633
128 -> 130 0.66338

Excited State 4: Singlet-A 4.0963 eV 302.68 nm f=0.0988 <S**2>=0.000
127 -> 130 0.66213
128 -> 130 -0.18246

Excited State 5: Singlet-A 4.3360 eV 285.94 nm f=0.0317 <S**2>=0.000
124 -> 130 -0.14121
127 -> 132 -0.22459
128 -> 131 0.32775
128 -> 132 0.52603

Excited State 6: Singlet-A 4.3614 eV 284.28 nm f=0.1386 <S**2>=0.000
127 -> 132 0.12543
128 -> 131 0.60456
128 -> 132 -0.27814

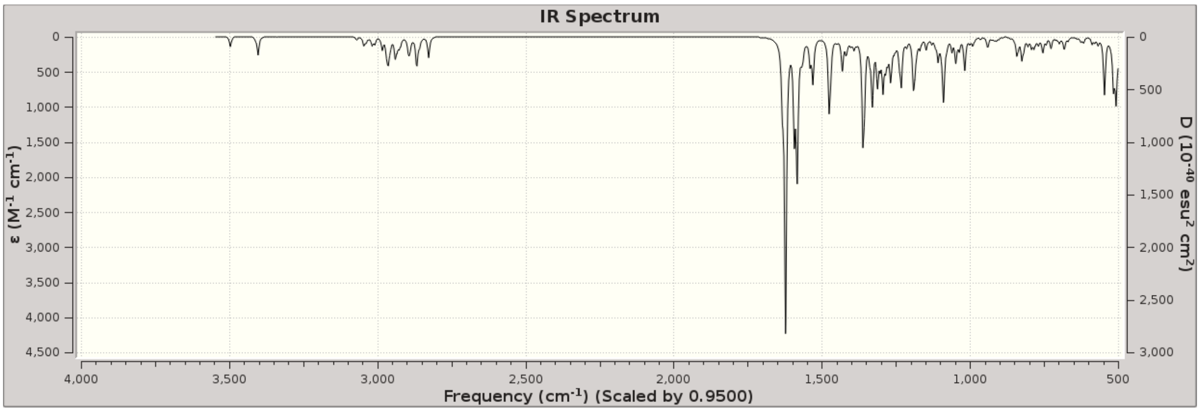

Figure S4. Calculated FTIR spectrum of probe A in water.

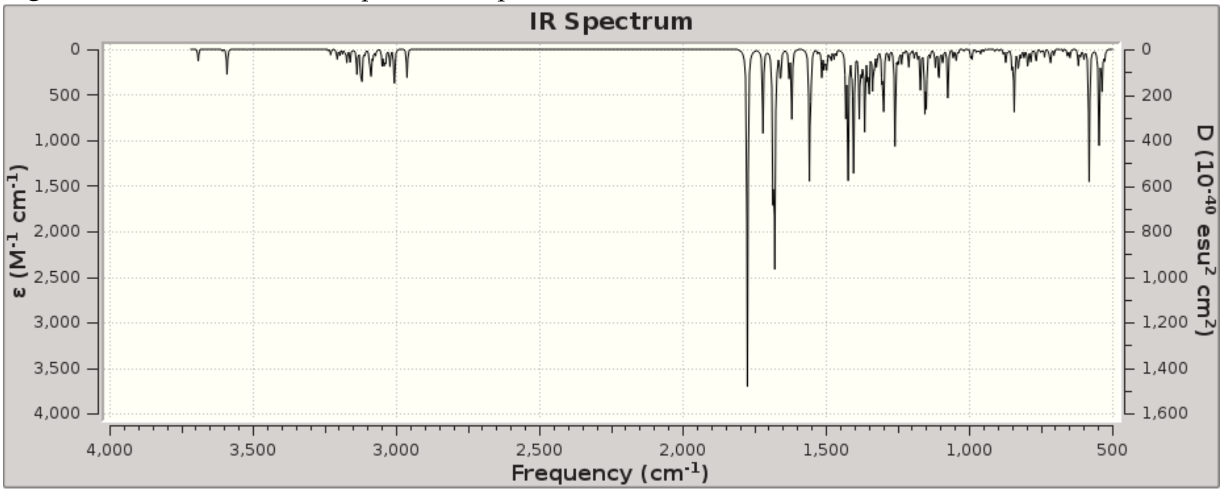

Figure S5. Calculated FTIR spectrum of probe A in gas phase without solution.

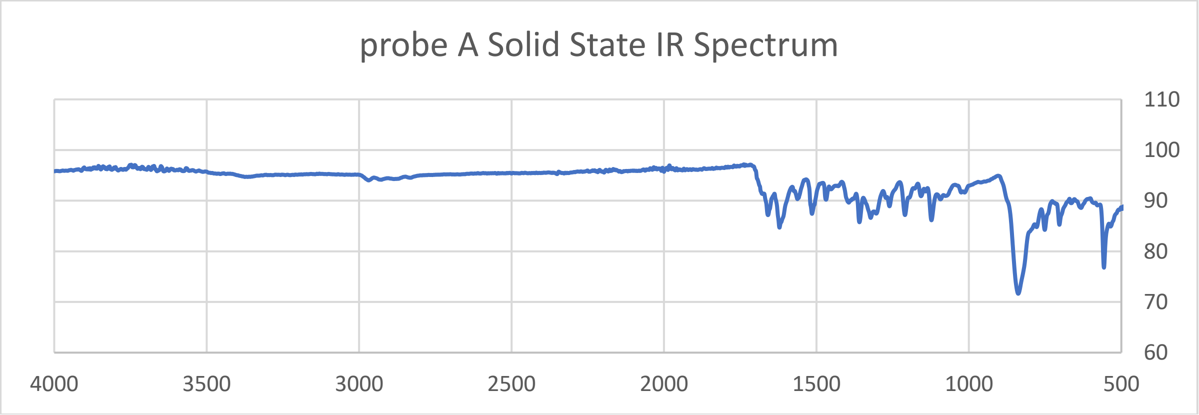

Figure S6. The solid state FTIR spectrum of probe A.

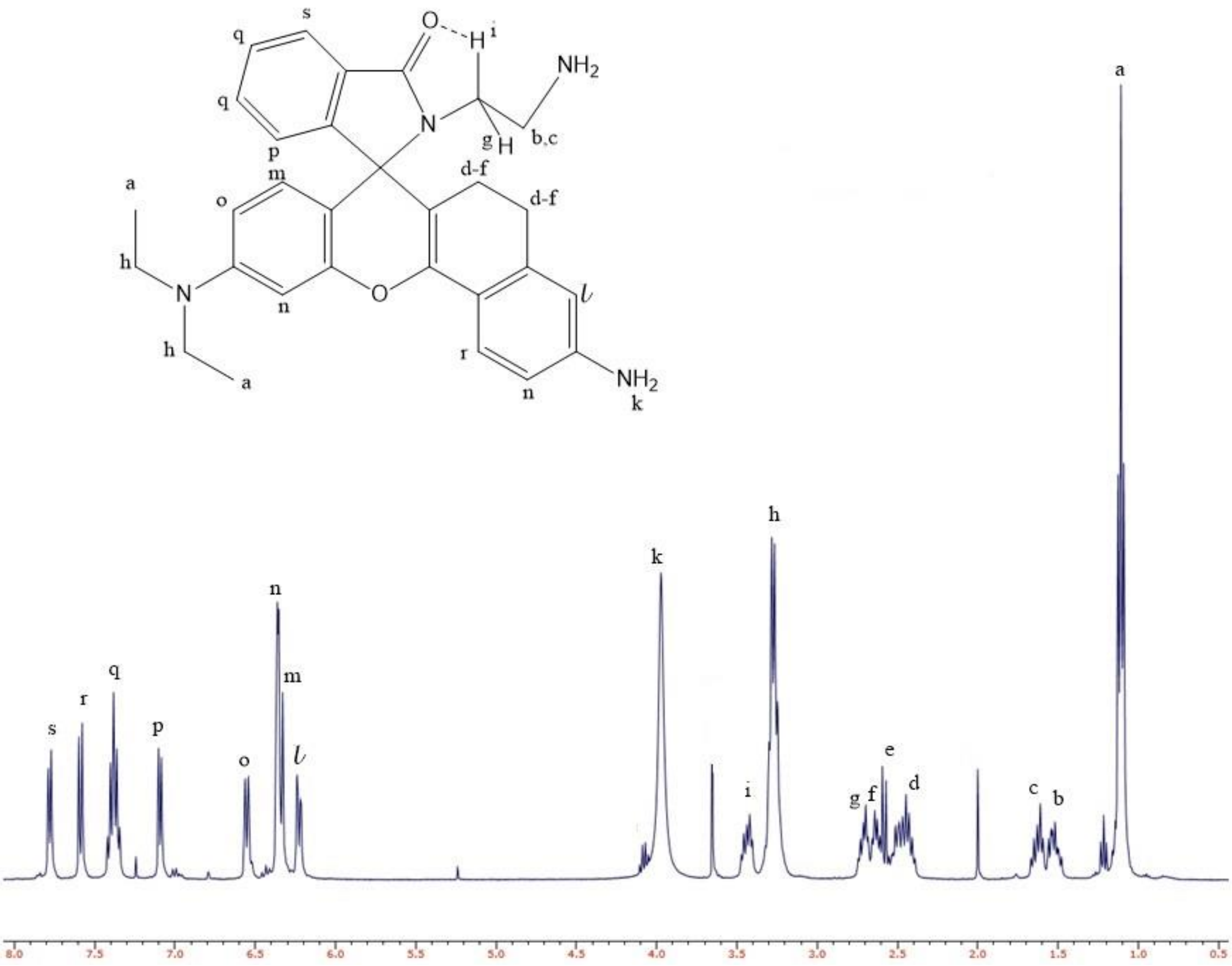

Figure S7. Tentative assignments for the proton NMR spectrum of probe A. This was based in part on the calculated spectrum shown below.

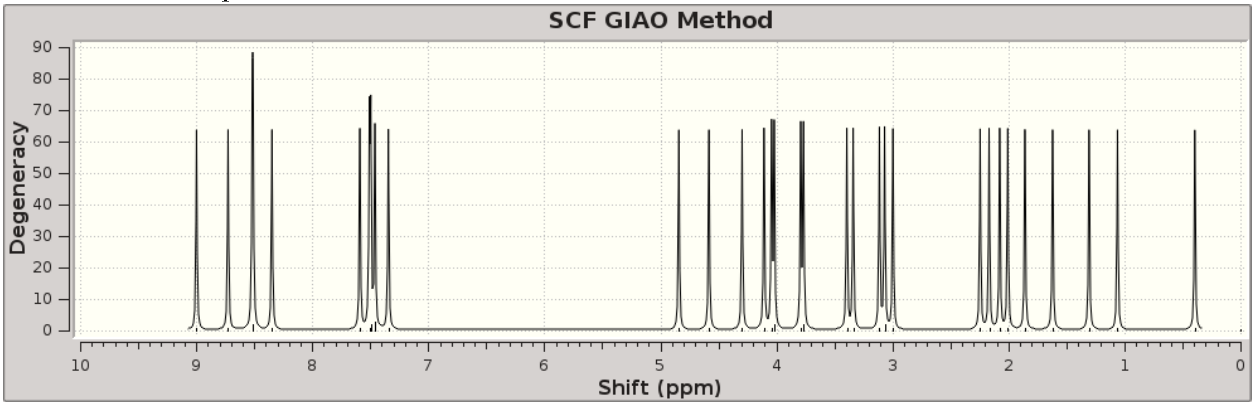

Figure S8. Calculated proton NMR spectrum of probe A.

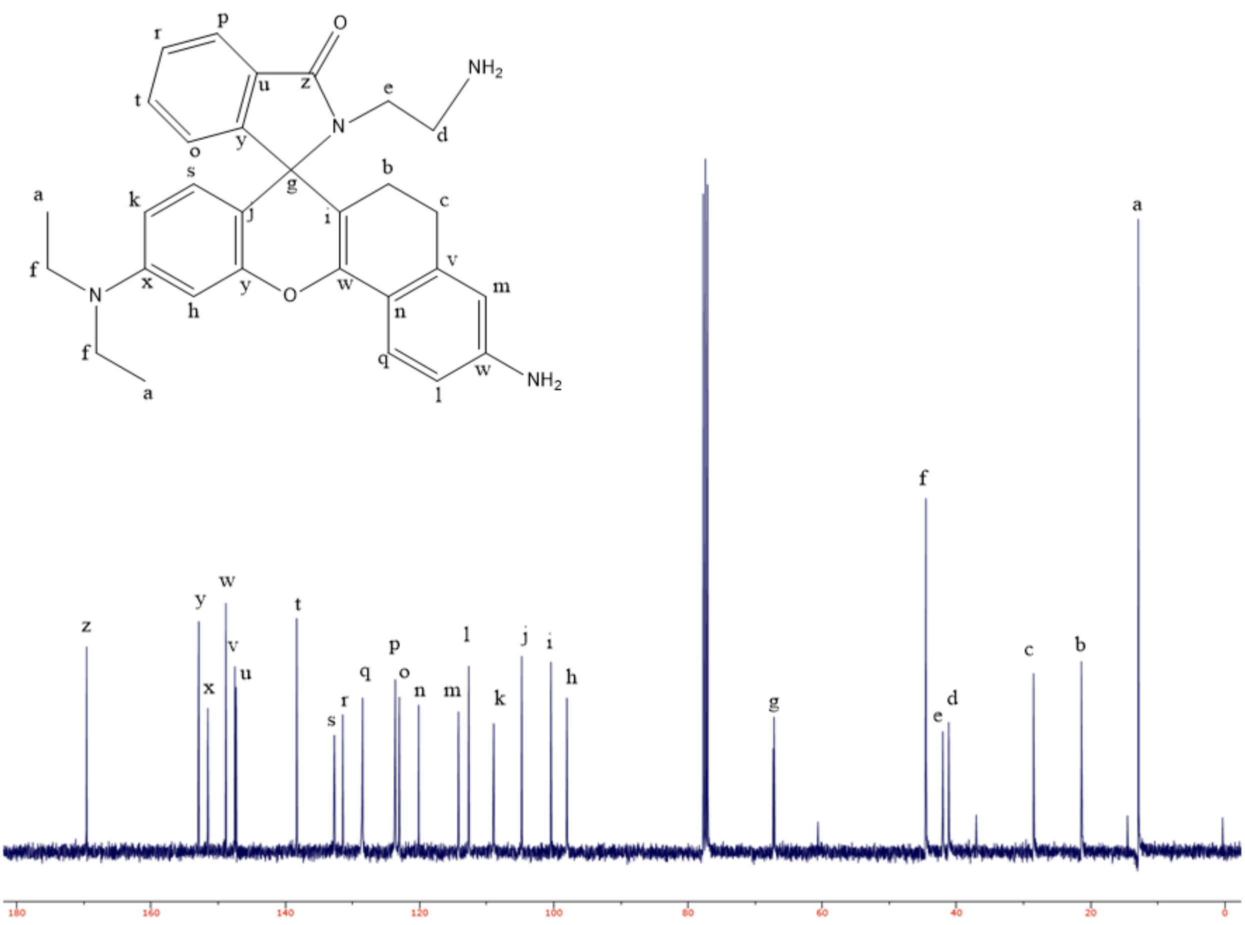

Figure S9. Tentative assignments for the 13C NMR spectrum of probe A.

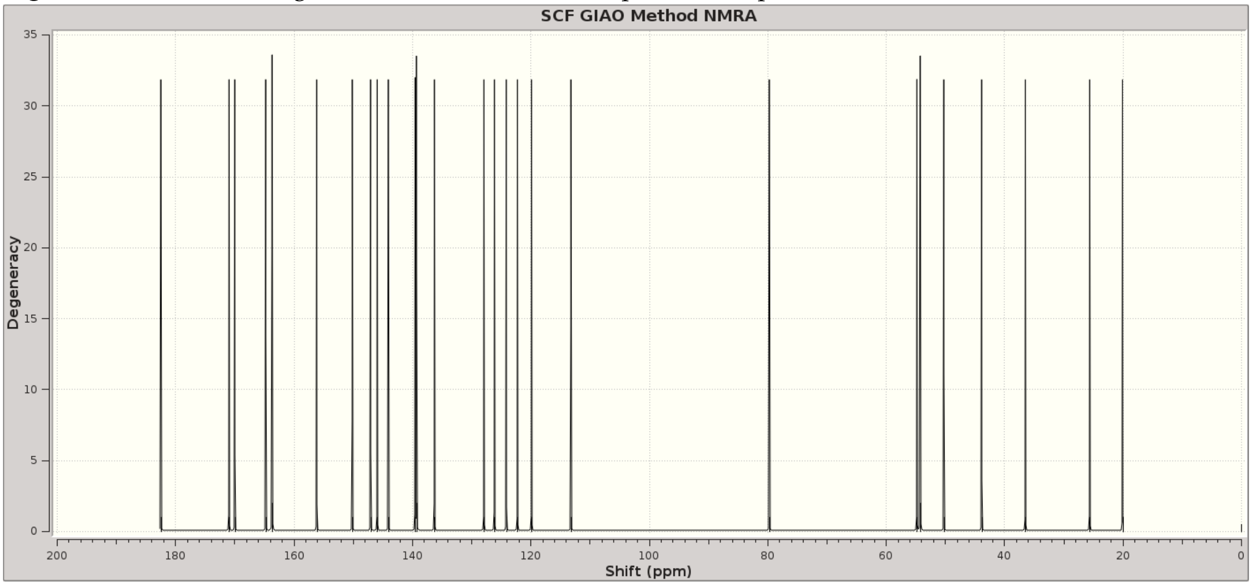

Figure S10. Calculated ^{13}C NMR spectrum for probe A

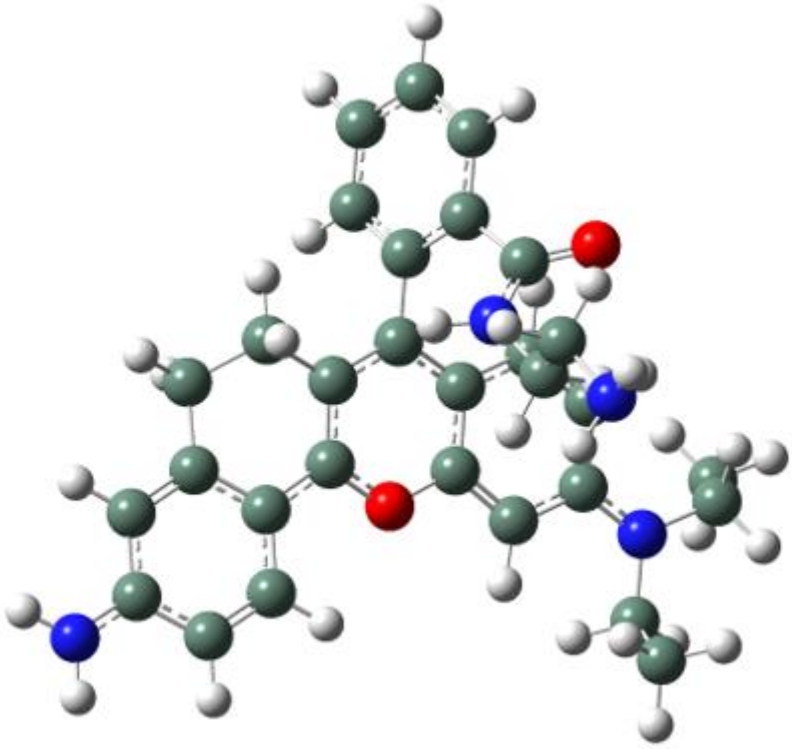

Figure S11. Drawing of probe AH+ with atoms represented as spheres of arbitrary size (H-white, C-grey, N-blue and O-red) using the GaussView[1] program.

Table S3. Atomic coordinates for probe AH+.

Row	Symbol	X	Y	Z	Row	Symbol	X	Y	Z
1	C	-0.26039	1.050589	-0.95814	20	C	3.597336	-4.11819	-0.18987
2	C	0.821269	0.14386	-0.8977	21	N	3.876892	-2.71371	-0.46238
3	C	0.5545	-1.20471	-0.57972	22	C	5.28693	-2.34928	-0.5479
4	O	-0.72794	-1.61468	-0.39897	23	C	5.826993	-2.40836	-1.97087
5	C	-1.7561	-0.76768	-0.48972	24	C	1.210894	2.742513	1.026583
6	C	-1.55862	0.592241	-0.76188	25	C	0.701268	3.278878	-0.27749
7	C	-3.03919	-1.33753	-0.25984	26	C	0.006806	2.487224	-1.20474
8	C	-4.19088	-0.57839	-0.59018	27	C	0.985246	4.60972	-0.58416
9	C	-4.00698	0.76637	-1.23346	28	C	0.569548	5.162879	-1.78806
10	C	-2.77468	1.476749	-0.68644	29	C	-0.11844	4.377477	-2.71041
11	C	2.179995	0.477675	-1.10216	30	C	-0.39389	3.047204	-2.42048
12	C	3.172798	-0.45108	-0.96676	31	C	0.691961	1.34652	2.976101
13	C	2.886598	-1.80827	-0.60982	32	O	2.365354	2.961574	1.386617
14	C	1.530873	-2.16084	-0.4303	33	C	0.505237	2.240886	4.197049
15	C	-3.19845	-2.63812	0.263644	34	N	0.883805	1.510389	5.402473
16	C	-4.44655	-3.16531	0.474949	35	N	-6.83175	-2.91708	0.368981
17	C	-5.60222	-2.40847	0.158681	36	H	-3.89272	0.620392	-2.31653
18	C	-5.44147	-1.11193	-0.37987	37	H	-4.89753	1.383854	-1.09352
19	C	3.429322	-4.40826	1.296648	38	H	-2.60566	2.414224	-1.21666
					39	H	-2.95124	1.741745	0.366288

40	H	2.43695	1.494356	-1.379	55	H	-0.90844	2.425161	-3.14716
41	H	4.193476	-0.14343	-1.15245	56	H	0.077085	0.445437	3.062434
42	H	1.22417	-3.16232	-0.15958	57	H	1.734509	1.030286	2.903728
43	H	-2.32065	-3.22359	0.515682	58	H	1.159987	3.111589	4.098054
44	H	-4.55601	-4.16277	0.890206	59	H	-0.52997	2.615938	4.214164
45	H	3.231333	-5.4717	1.456898	60	H	0.893106	2.134978	6.201118
46	H	4.335295	-4.14719	1.850719	61	H	0.19161	0.797863	5.611578
47	H	2.59936	-3.84044	1.72529	62	H	-7.66184	-2.39639	0.141673
48	H	2.717279	-4.42831	-0.75843	63	H	-6.96122	-3.83996	0.747463
49	H	4.430053	-4.69619	-0.59815	64	N	0.332474	2.012537	1.742513
50	H	5.435615	-1.36205	-0.10554	65	H	-0.62698	1.973968	1.437767
51	H	5.834509	-3.04317	0.094916	66	H	-6.32334	-0.53144	-0.63824
52	H	6.88973	-2.1508	-1.98559	67	H	0.786173	6.203364	-2.01023
53	H	5.71854	-3.41282	-2.38962	68	H	-0.4346	4.79831	-3.66008
54	H	5.299952	-1.71249	-2.62902	69	H	1.53378	5.207187	0.137266

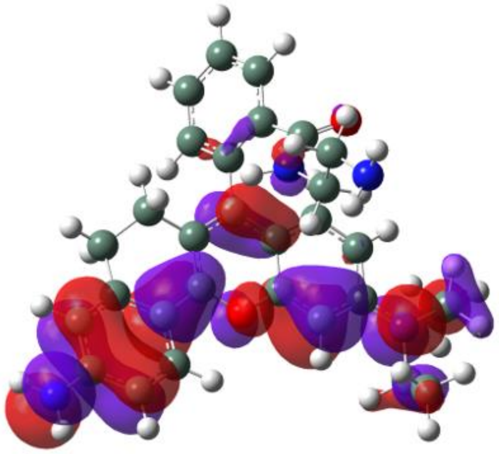

AH+-**127**

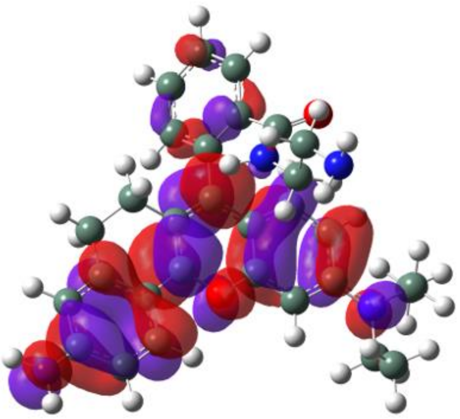

AH+-**128**

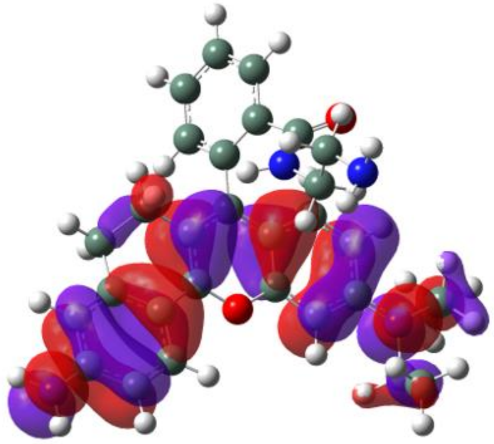

AH+-**129**

Figure S12. LCAO for orbitals 127, 128 and 129 in probe AH+.

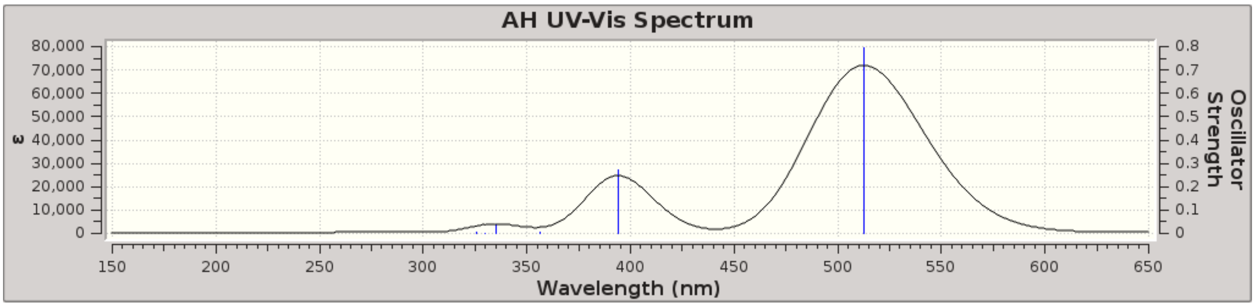

Figure S13. Calculated UV-Vis spectrum for probe AH+.

Table S4. Excitation Energies and Oscillator Strengths for AH^{+}.

Excited State 1: Singlet-A 2.4174 eV 512.88 nm f=0.7947 <S**2>=0.000
128 ->129 0.70239
This state for optimization and/or second-order correction.
Total Energy, E(TD-HF/TD-DFT) = -1531.45482165
Copying the excited state density for this state as the 1-particle RhoCI density.

Excited State 2: Singlet-A 3.1442 eV 394.33 nm f=0.2693 <S**2>=0.000
127 ->129 0.68665

Excited State 3: Singlet-A 3.4815 eV 356.12 nm f=0.0072 <S**2>=0.000
125 ->129 -0.14295
126 ->129 0.68938

Excited State 4: Singlet-A 3.7001 eV 335.08 nm f=0.0348 <S**2>=0.000
125 ->129 0.65144
126 ->129 0.14091
128 ->130 -0.18079

Excited State 5: Singlet-A 3.7578 eV 329.94 nm f=0.0007 <S**2>=0.000
124 ->129 0.68799
125 ->129 0.11533

Excited State 6: Singlet-A 3.8041 eV 325.92 nm f=0.0043 <S**2>=0.000
123 ->129 0.67045
128 ->130 0.15804

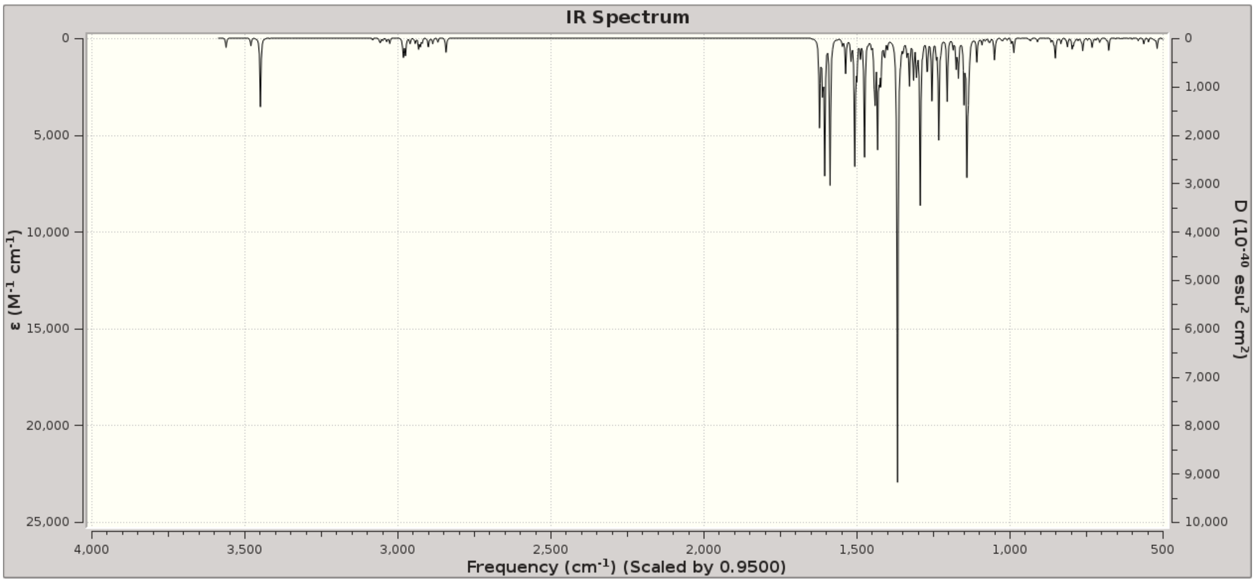

Figure S14. Calculated FTIR spectrum of probe AH^{+} in water.

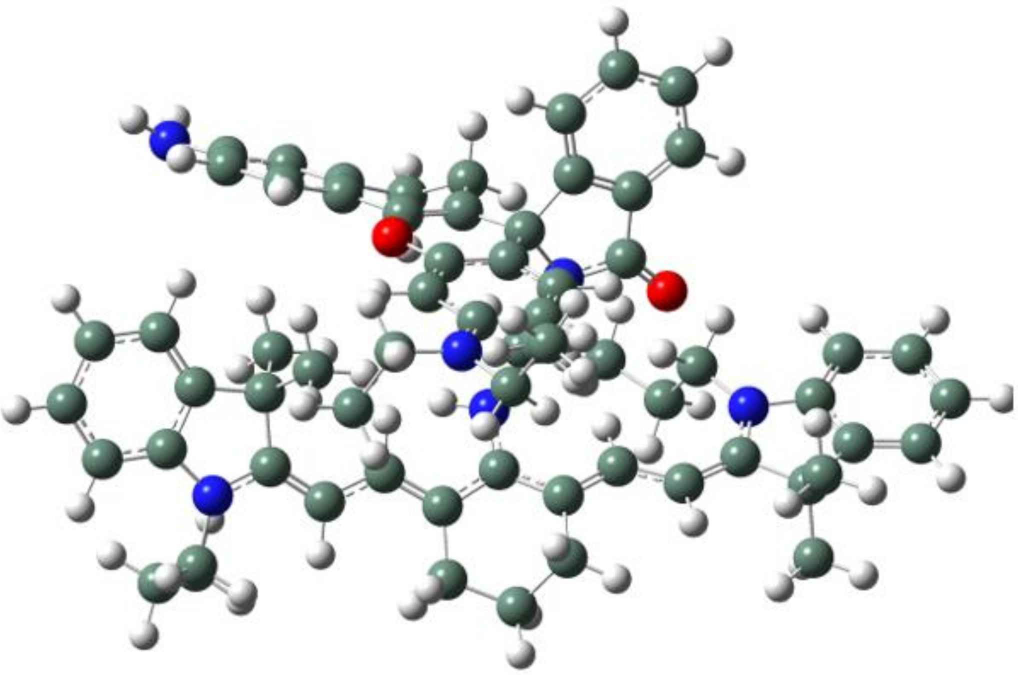

Figure S15. Drawing of probe B^+ with atoms represented as spheres of arbitrary size (H-white, C-grey, N-blue and O-red) using the GaussView[1] program.

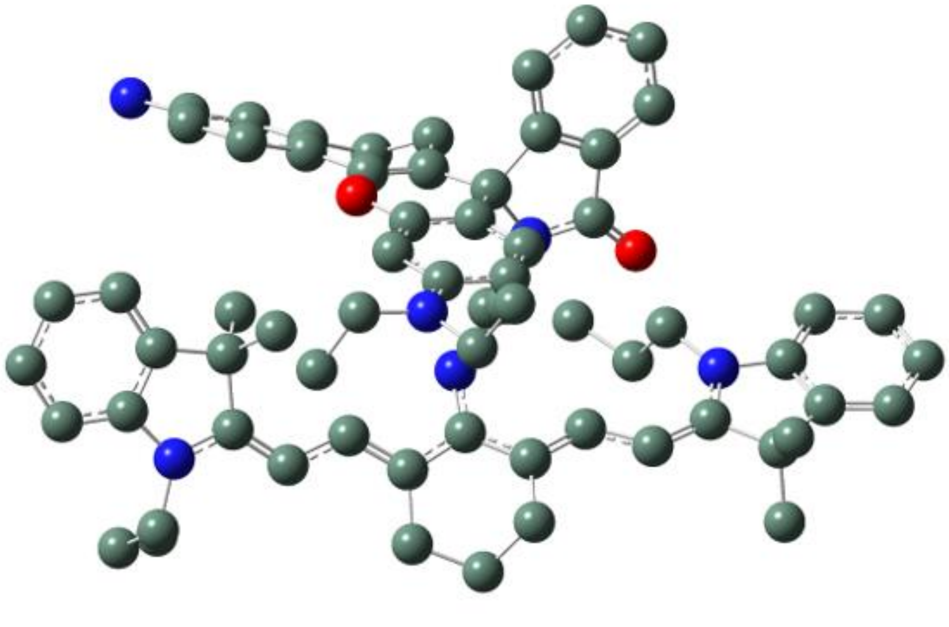

Figure S16. Drawing of probe B^+ with atoms represented as spheres of arbitrary size (C-grey, N-blue and O-red) using the GaussView[1] program. H-atoms are not depicted for clarity

Table S5. Atomic coordinates for probe B^+.

Row	Symbol	X	Y	Z	Row	Symbol	X	Y	Z
1	C	-2.5084	1.614571	-3.57082	2	C	-2.9292	2.163827	-2.35054
					3	C	-1.93316	2.414487	-1.31621

4	C	-0.6141	2.428276	-1.56499	50	C	5.742366	-1.75951	0.531924
5	C	-0.15348	2.248161	-2.98459	51	C	6.974658	-1.7406	1.431801
6	C	0.395383	2.729354	-0.49597	52	C	-3.97911	-2.78111	0.359968
7	C	-0.28164	2.874324	0.842347	53	C	-4.24395	-1.43737	-0.31781
8	C	-1.66101	2.807876	0.973618	54	C	7.274385	-3.1385	1.984806
9	C	-2.29848	2.875785	2.202693	55	H	6.472879	-3.47837	2.644297
10	C	-1.55845	3.024445	3.385617	56	H	7.389139	-3.86308	1.176033
11	C	-0.14968	3.114812	3.256355	57	H	8.201996	-3.11547	2.561589
12	C	0.449534	3.035927	2.018948	58	C	6.793482	-0.73203	2.573651
13	O	-2.49262	2.652003	-0.09692	59	H	6.561095	0.261672	2.185102
14	C	1.24942	3.927821	-0.8494	60	H	5.987305	-1.04104	3.242774
15	C	2.585135	3.569291	-0.91419	61	H	7.715079	-0.66805	3.157042
16	C	0.849883	5.229016	-1.0921	62	C	-3.66145	-0.28333	0.509416
17	C	3.573917	4.492758	-1.21493	63	H	-3.99992	-0.33975	1.546166
18	C	1.832942	6.16554	-1.39752	64	H	-2.57082	-0.29346	0.504746
19	H	-0.19743	5.510322	-1.04585	65	H	-3.98951	0.666928	0.089749
20	C	3.181667	5.803576	-1.45768	66	C	-3.75899	-1.38725	-1.77392
21	C	2.701784	2.125451	-0.61554	67	H	-2.6717	-1.38425	-1.84879
22	O	3.718075	1.440048	-0.54374	68	H	-4.14168	-2.23828	-2.34098
23	N	1.434703	1.680492	-0.4222	69	H	-4.12114	-0.46889	-2.24077
24	C	-1.40934	3.093004	5.841908	70	N	6.099458	-1.33993	-0.70374
25	C	-4.28605	2.424399	-2.15849	71	N	-5.16437	-3.33215	0.711594
26	N	-2.16904	3.075925	4.60622	72	C	7.474001	-1.07871	-0.77012
27	H	1.549787	7.1948	-1.59284	73	C	8.045755	-1.29204	0.478165
28	H	3.925932	6.555387	-1.69808	74	C	8.223972	-0.67319	-1.86281
29	H	4.61641	4.195491	-1.25943	75	C	9.396474	-1.08701	0.668401
30	C	-1.03958	4.49828	6.301186	76	C	9.58828	-0.47004	-1.66193
31	H	-2.01575	2.597769	6.605262	77	H	7.783768	-0.523	-2.8415
32	H	-0.51539	2.474344	5.728538	78	C	10.17262	-0.67023	-0.41514
33	H	-1.93751	5.101545	6.459594	79	H	9.851563	-1.24781	1.640827
34	H	-0.42053	5.005615	5.557281	80	H	10.20245	-0.15278	-2.49821
35	H	-0.48377	4.463446	7.242199	81	H	11.23685	-0.50559	-0.28588
36	H	0.474295	3.254953	4.128871	82	C	-5.74829	-1.3786	-0.28469
37	H	1.53235	3.097659	1.95576	83	C	-6.61605	-0.41139	-0.74637
38	C	1.132458	0.325722	-0.0511	84	C	-6.2381	-2.52109	0.332347
39	N	0.795969	-1.96033	-0.99575	85	C	-7.98826	-0.60604	-0.57794
40	C	0.88457	-2.63359	0.193365	86	H	-6.2396	0.481424	-1.23414
41	C	2.094174	-2.68174	0.939841	87	C	-7.59525	-2.73927	0.511799
42	C	-0.28669	-3.27939	0.681058	88	C	-8.46541	-1.75587	0.044452
43	C	1.995323	-3.27494	2.31711	89	H	-8.68716	0.142601	-0.93525
44	C	-0.18577	-4.35833	1.730307	90	H	-7.9779	-3.63305	0.990136
45	C	1.239955	-4.59902	2.214595	91	H	-9.53423	-1.89561	0.168018
46	C	3.279198	-2.26639	0.371019	92	C	5.233559	-1.19404	-1.85537
47	C	4.515134	-2.15453	1.022474	93	H	4.31389	-0.71258	-1.52072
48	C	-1.52608	-2.8216	0.272997	94	H	5.703896	-0.47801	-2.53103
49	C	-2.76049	-3.36718	0.636427	95	C	4.988382	-2.51526	-2.57515

96	H	5.939065	-2.87177	-2.98502	123	C	-4.80279	1.578398	-4.36043
97	H	4.663396	-3.27263	-1.85459	124	C	-5.21302	2.14108	-3.1457
98	C	3.954956	-2.36221	-3.68206	125	N	-5.72649	1.226042	-5.32563
99	H	2.975285	-2.09021	-3.27846	126	H	-6.60847	1.715474	-5.29037
100	H	4.247567	-1.58473	-4.39376	127	H	-5.37156	1.147811	-6.26721
101	H	3.833229	-3.2945	-4.23782	128	H	-4.61977	2.857331	-1.22212
102	C	-5.36775	-4.605	1.380121	129	H	-6.26467	2.357027	-2.98213
103	H	-4.46526	-4.84959	1.939931	130	C	-1.05381	1.280295	-3.74416
104	H	-6.16093	-4.45911	2.11737	131	H	-0.90104	0.260255	-3.36344
105	C	-5.73203	-5.72657	0.411701	132	H	-0.79007	1.256157	-4.80427
106	H	-6.65593	-5.46129	-0.11265	133	H	0.881202	1.893432	-3.00183
107	H	-5.96204	-6.6134	1.011366	134	H	-0.14546	3.223075	-3.49188
108	C	-4.63397	-6.03981	-0.59546	135	H	-2.7608	-4.31042	1.167993
109	H	-4.38758	-5.16525	-1.20324	136	H	-0.81358	-4.06589	2.581576
110	H	-3.71773	-6.36682	-0.09593	137	H	-0.61771	-5.29149	1.348588
111	H	-4.94666	-6.83659	-1.27454	138	C	-3.61306	3.046229	4.732381
112	C	1.245093	-0.60009	-1.25849	139	H	1.221498	-5.11334	3.179513
113	H	0.117141	0.304159	0.350951	140	H	1.775079	-5.25006	1.514774
114	H	1.814597	0.009806	0.740613	141	H	2.986443	-3.43976	2.740804
115	H	-0.01339	-2.21929	-1.53712	142	H	1.465816	-2.59732	2.999345
116	H	0.605206	-0.21991	-2.05298	143	H	-3.87116	3.564316	5.659864
117	H	2.265701	-0.56177	-1.64428	144	C	-4.18843	1.63396	4.745429
118	H	-3.37555	2.777521	2.208573	145	H	-4.05856	3.639059	3.928914
119	C	-3.43767	1.326295	-4.5531	146	H	-3.78482	1.060248	5.583992
120	H	-1.5446	-1.92873	-0.33873	147	H	-3.94401	1.099541	3.824377
121	H	3.228667	-2.02492	-0.67936	148	H	-5.27681	1.661303	4.845689
122	H	4.535378	-2.39938	2.078213	149	H	-3.104	0.889381	-5.49073

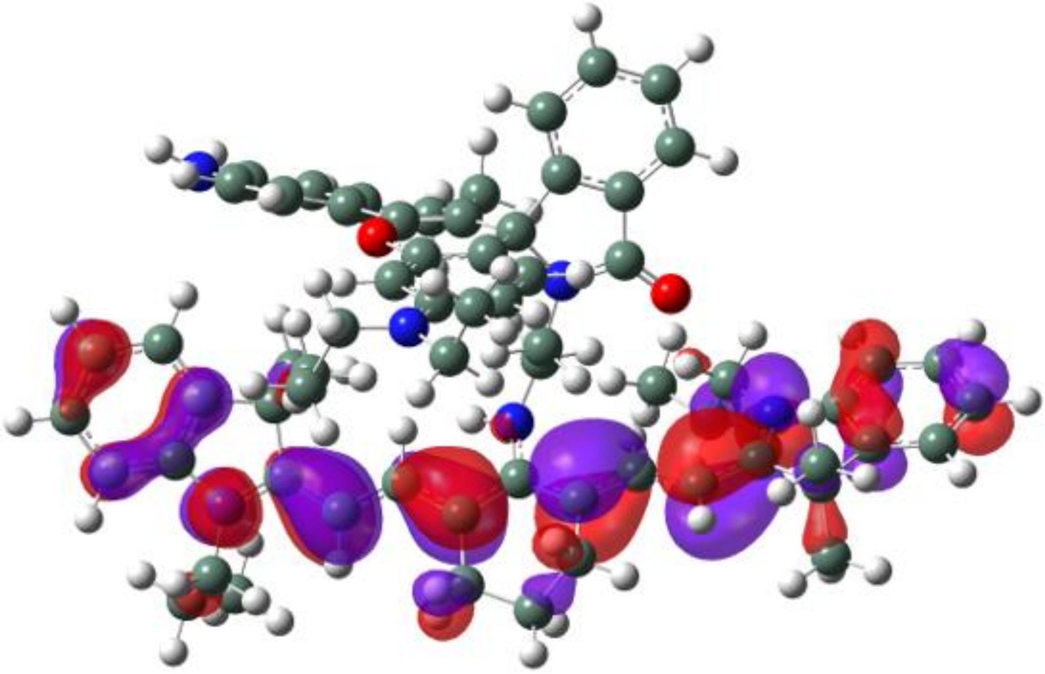

B⁺-264

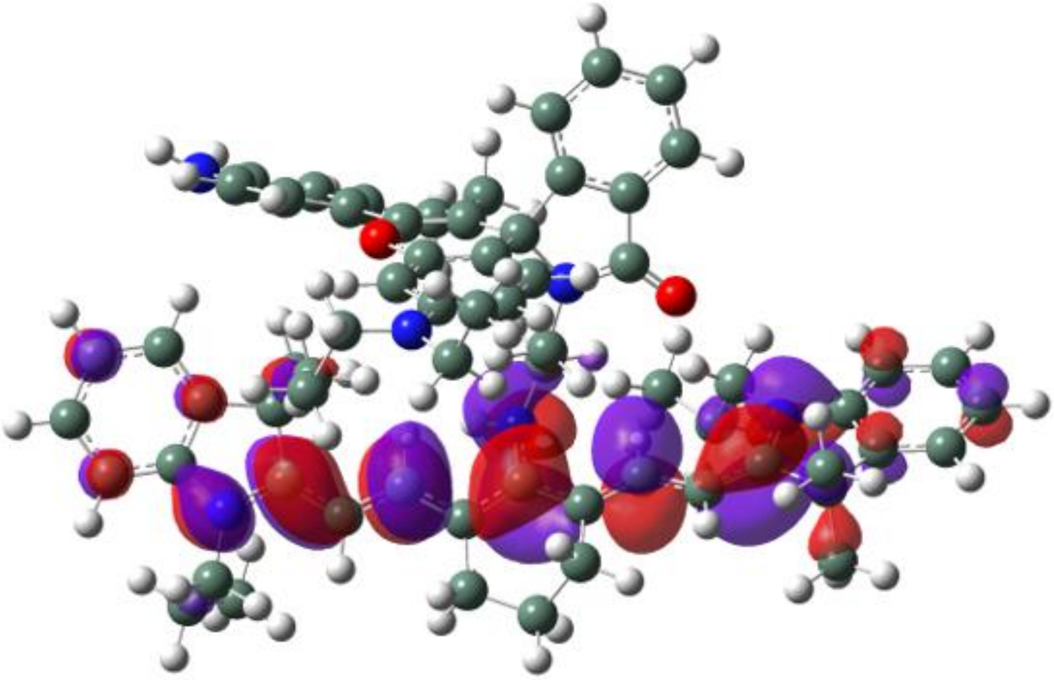

B⁺-265

Figure S17. LCAO for orbitals 264 and 265 in probe B^+.

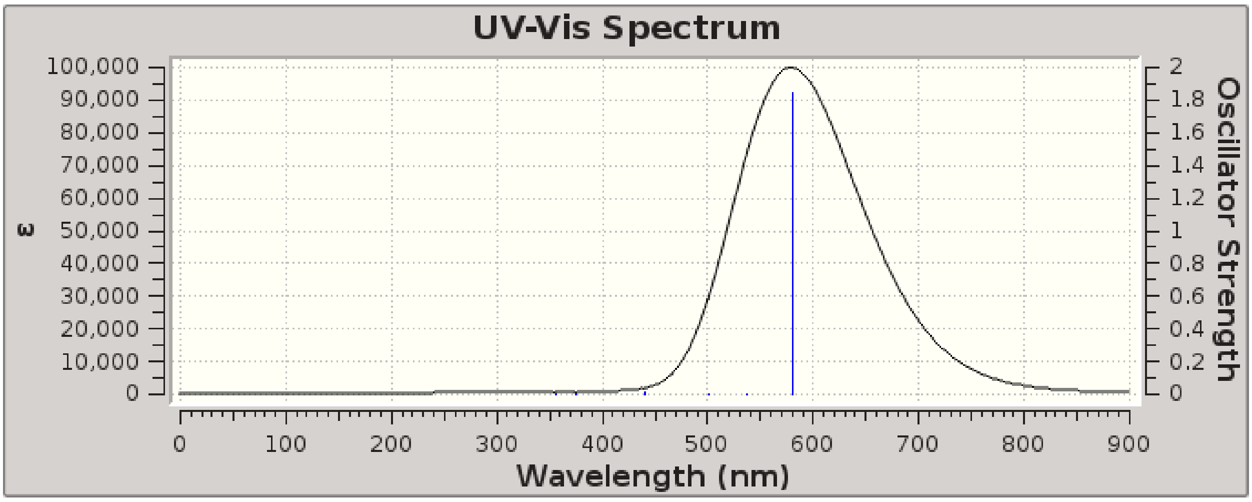

Figure S18. Calculated UV-Vis spectrum for probe B^+. This represents a HOMO-LUMO transition.

Table S6. Excitation Energies and Oscillator Strengths for B^+.

Excited State 1: Singlet-A 2.1382 eV 579.86 nm f=1.8426 <S**2>=0.000
264 -> 265 0.70533
This state for optimization and/or second-order correction.
Total Energy, E(TD-HF/TD-DFT) = -3037.42763374
Copying the excited state density for this state as the 1-particle RhoCI density.

Excited State 2: Singlet-A 2.3061 eV 537.64 nm f=0.0005 <S**2>=0.000
263 -> 265 0.70655

Excited State 3: Singlet-A 2.4750 eV 500.95 nm f=0.0035 <S**2>=0.000
262 -> 265 0.70573

Excited State 4: Singlet-A 2.8158 eV 440.31 nm f=0.0119 <S**2>=0.000
261 -> 265 0.69592

Excited State 5: Singlet-A 3.3049 eV 375.15 nm f=0.0000 <S**2>=0.000
260 -> 265 0.70574

Excited State 6: Singlet-A 3.4857 eV 355.70 nm f=0.0020 <S**2>=0.000
264 -> 266 0.70403

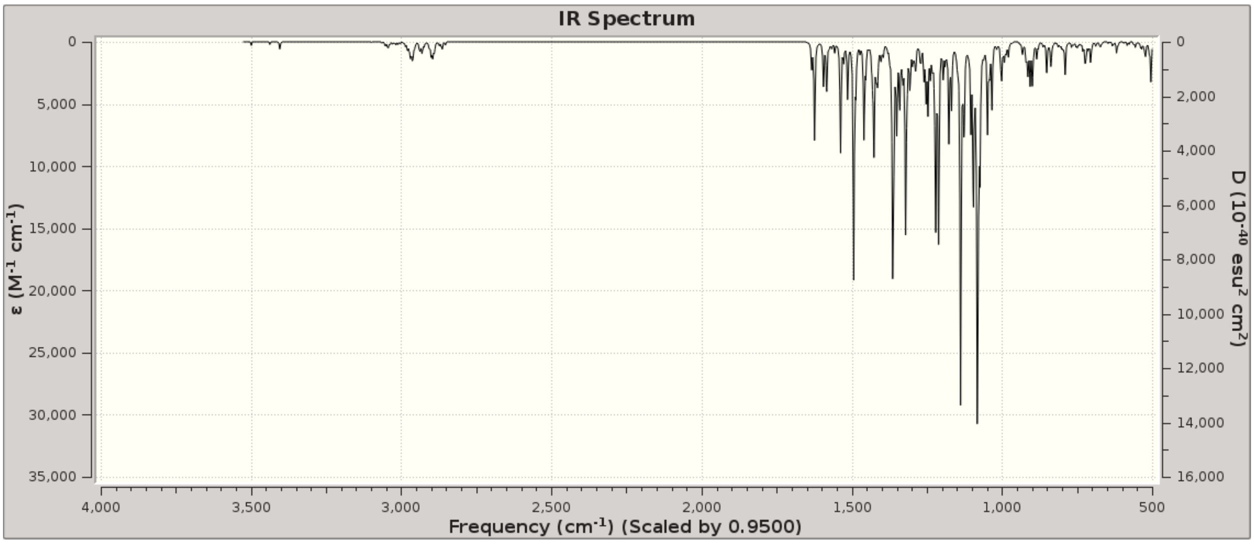

Figure S19. Calculated FTIR spectrum of probe B^+ in water.

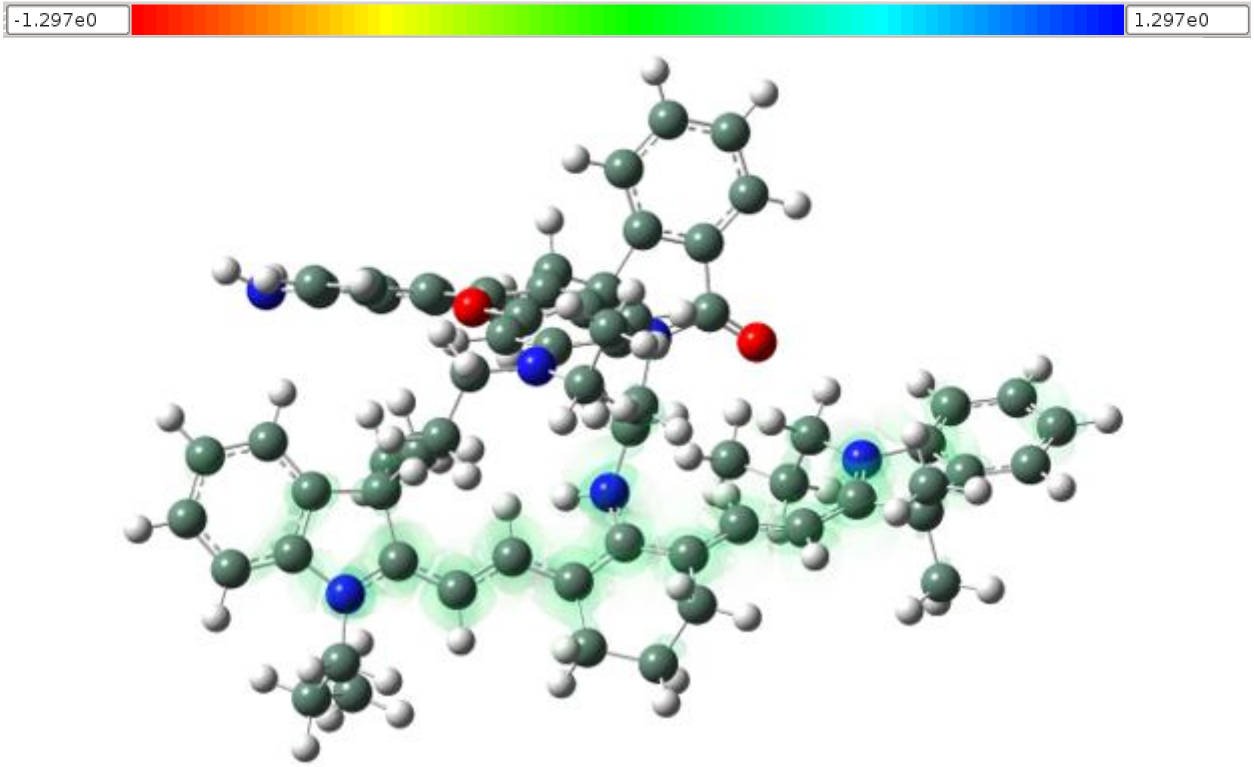

Figure S20. Current density difference plot for probe B^+ obtained by subtracting the SCF (ground state) density from the CI (excited state) density using the Cubegen program in GaussView.

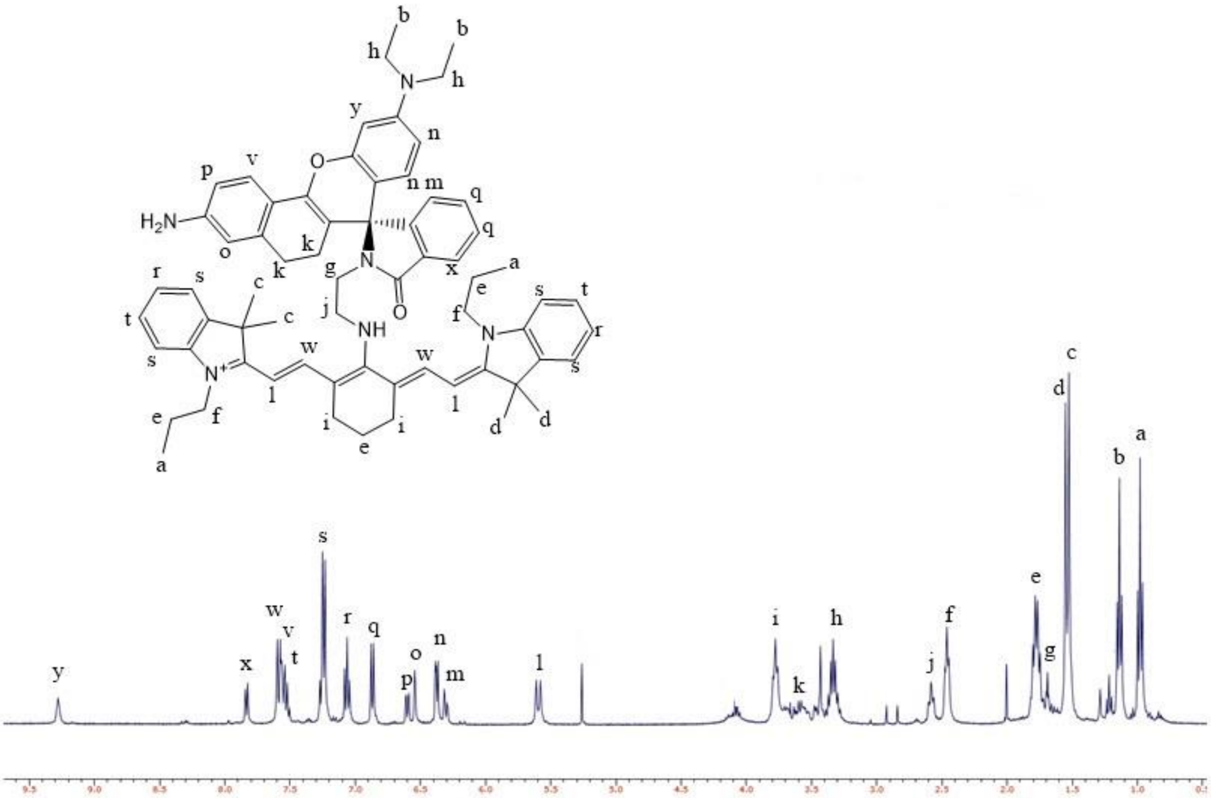

Figure S21. Tentative assignments for the ^{1}H NMR spectrum of probe B^{+}. This was based in part on the calculated spectrum shown below.

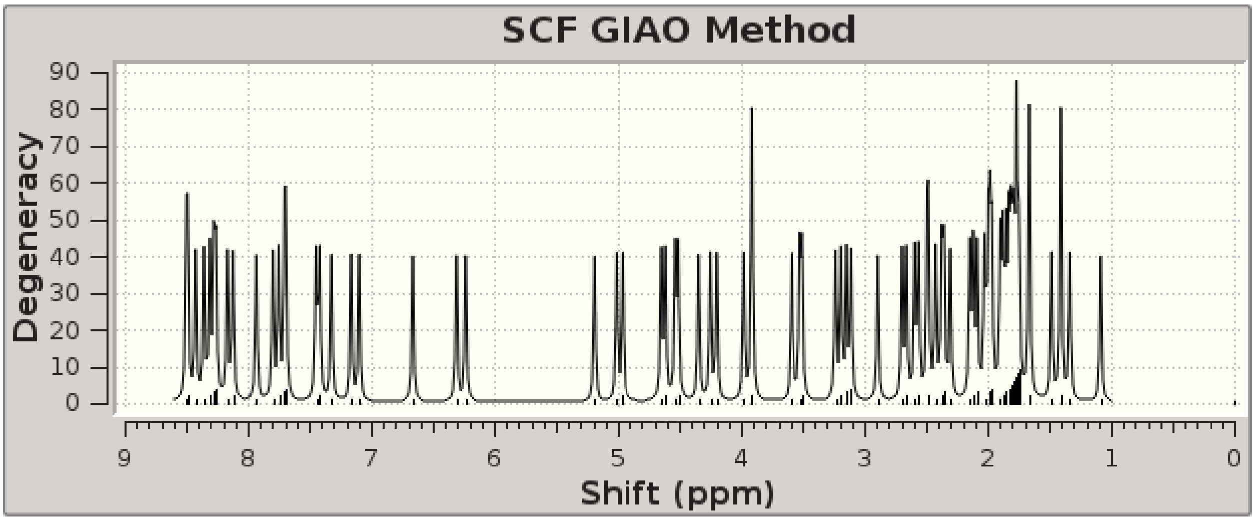

Figure S22. Calculated ^{1}H NMR spectrum of probe B^{+}.

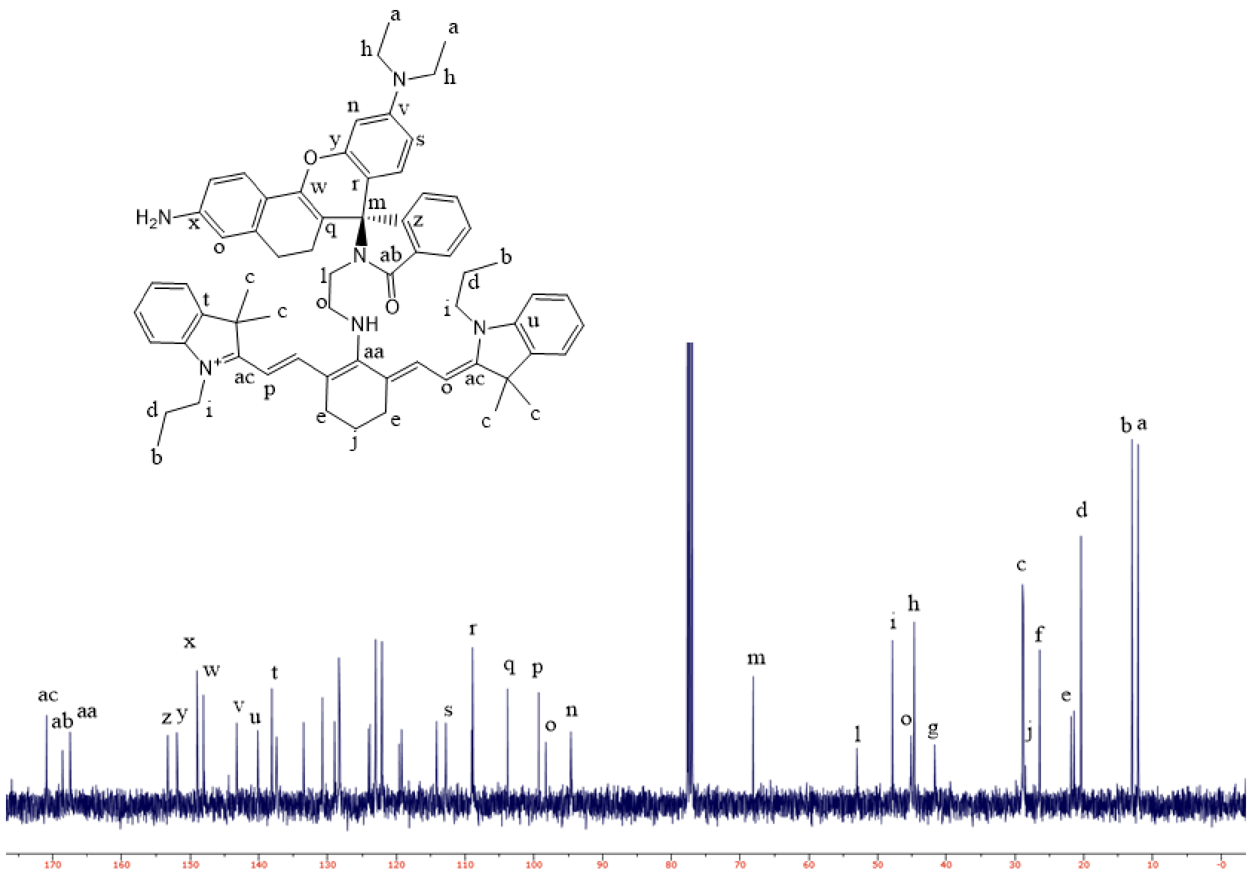

Figure S23. Partial assignments of the ^{13}C NMR spectrum of probe B^+.

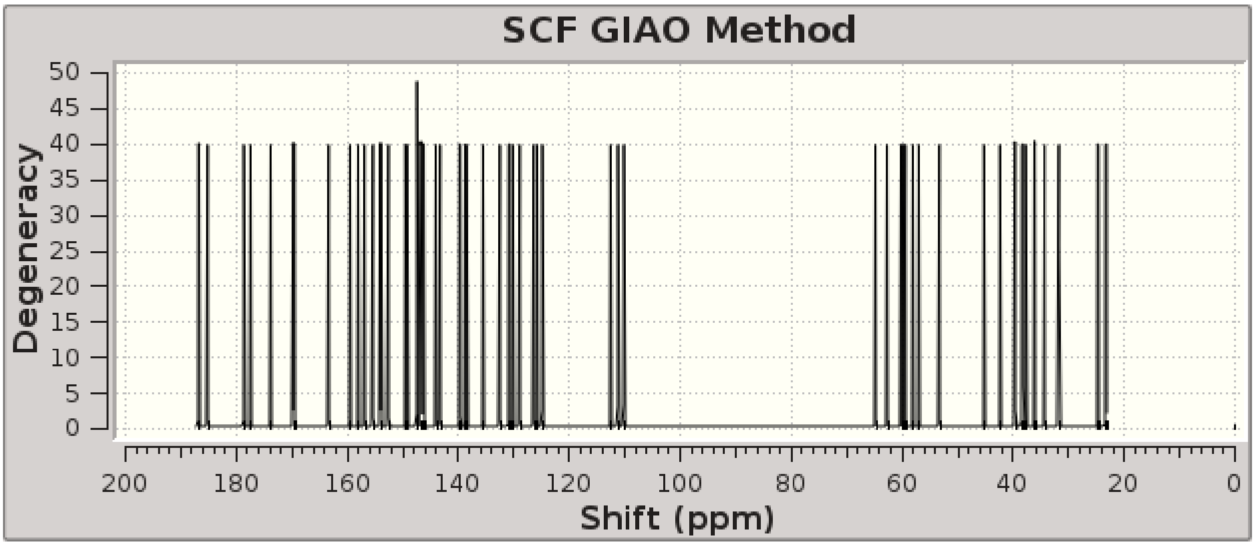

Figure S24. Calculated ^{13}C NMR spectrum of probe B^+.

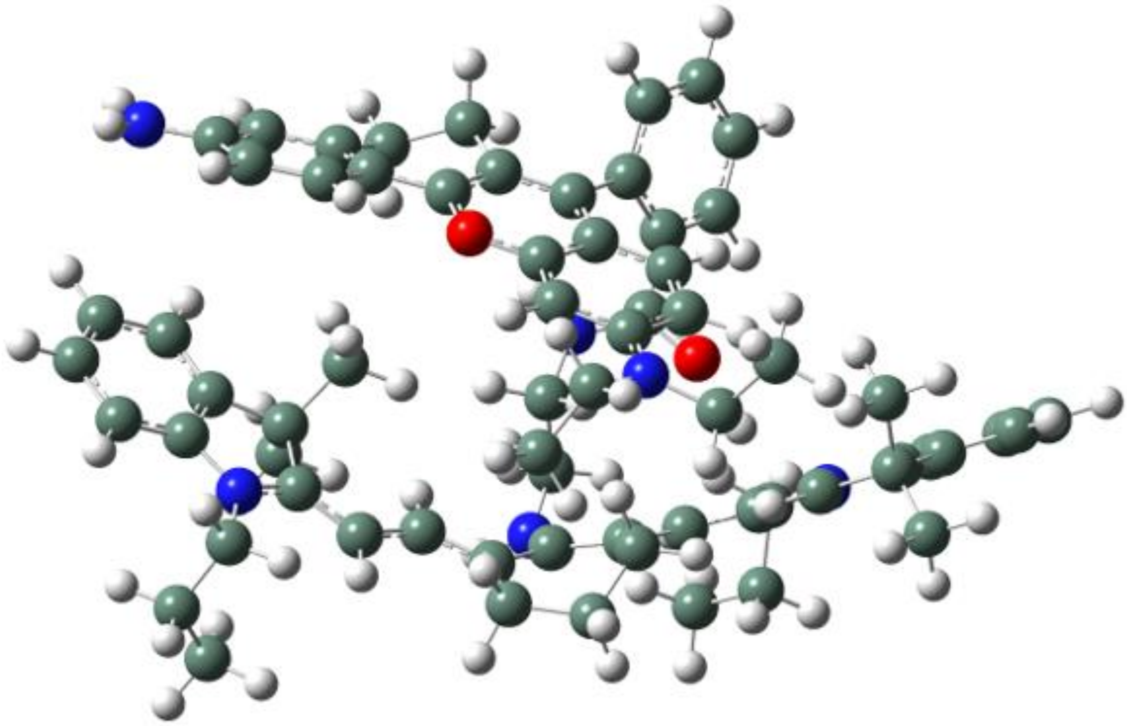

Figure S25. Drawing of probe BH^{2+} with atoms represented as spheres of arbitrary size (H-white, C-grey, N-blue and O-red) using the GaussView [1] program.

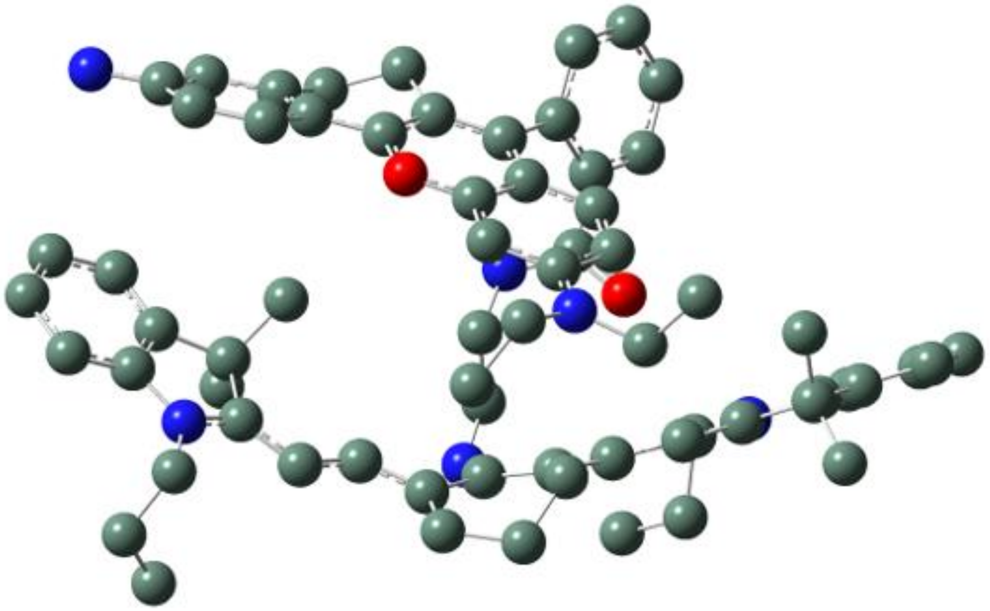

Figure S26. Drawing of probe BH^{2+} with atoms represented as spheres of arbitrary size (C-grey, N-blue and O-red) using the GaussView [1] program. H-atoms are not depicted for clarity

Table S7. Atomic coordinates for probe BH^{2+}.

Row	Symbol	X	Y	Z	Row	Symbol	X	Y	Z
1	C	-3.77906	4.110412	-0.35036	45	C	0.762698	-4.71518	0.722567
2	C	-3.47745	3.265797	0.742288	46	C	2.865643	-2.16545	-0.8139
3	C	-2.11521	2.950591	0.998357	47	C	3.991279	-1.98009	-0.00606
4	C	-1.04492	3.681396	0.473651	48	C	-1.89382	-2.71541	-1.1102
5	C	-1.39816	4.882518	-0.35928	49	C	-3.16225	-3.12478	-0.67412
6	C	0.251358	3.268186	0.758741	50	C	5.191974	-1.35747	-0.28211
7	C	0.455824	2.130962	1.57294	51	C	6.258833	-1.21319	0.798512
8	C	-0.67036	1.439242	2.062177	52	C	-4.29019	-2.33771	-0.76012
9	C	-0.59701	0.302628	2.827638	53	C	-4.39692	-0.91723	-1.31409
10	C	0.662373	-0.22772	3.167072	54	C	6.742955	-2.58897	1.273236
11	C	1.817168	0.468483	2.695339	55	H	5.934991	-3.14058	1.759188
12	C	1.711844	1.600768	1.942795	56	H	7.114821	-3.18322	0.436022
13	O	-1.91784	1.879109	1.76496	57	H	7.553252	-2.46514	1.995537
14	C	1.414608	4.008633	0.211777	58	C	5.73292	-0.38784	1.978994
15	C	2.318936	3.414141	-0.68032	59	H	5.329683	0.569197	1.642477
16	C	1.642189	5.317604	0.634212	60	H	4.950292	-0.93197	2.511319
17	C	3.429558	4.124128	-1.12297	61	H	6.544275	-0.19441	2.684386
18	C	2.743329	6.025192	0.175465	62	C	-3.53151	0.058542	-0.50896
19	H	0.956892	5.774726	1.339941	63	H	-3.81305	0.045934	0.544973
20	C	3.637689	5.430268	-0.70787	64	H	-2.47323	-0.18973	-0.57347
21	C	2.157774	1.988262	-1.09275	65	H	-3.67662	1.071596	-0.88787
22	O	3.072158	1.18278	-0.96421	66	C	-4.09129	-0.84813	-2.81666
23	N	0.931649	1.642821	-1.53993	67	H	-3.03978	-1.03152	-3.03813
24	C	2.0615	-1.96552	4.194833	68	H	-4.69033	-1.57563	-3.3678
25	C	-4.52895	2.699661	1.488726	69	H	-4.33917	0.149207	-3.18679
26	N	0.775309	-1.34209	3.917816	70	N	5.641819	-0.78386	-1.41852
27	H	2.906769	7.042506	0.514424	71	N	-5.50764	-2.71489	-0.29939
28	H	4.498289	5.983564	-1.06783	72	C	6.932752	-0.26633	-1.24634
29	H	4.12512	3.640204	-1.80016	73	C	7.345267	-0.48209	0.062675
30	C	2.745004	-1.4036	5.433353	74	C	7.740622	0.369702	-2.17571
31	H	1.877804	-3.03615	4.312171	75	C	8.585624	-0.0482	0.48211
32	H	2.699849	-1.87029	3.31571	76	C	8.992364	0.804566	-1.74367
33	H	2.118636	-1.53629	6.318816	77	H	7.429845	0.524711	-3.20205
34	H	2.95127	-0.33655	5.32253	78	C	9.414293	0.603988	-0.43302
35	H	3.692345	-1.9193	5.607782	79	H	8.915454	-0.21063	1.503551
36	H	2.804228	0.117846	2.960199	80	H	9.64757	1.306486	-2.44793
37	H	2.613389	2.109477	1.625073	81	H	10.39338	0.95249	-0.12259
38	C	0.459331	0.282367	-1.4422	82	C	-5.85135	-0.60816	-1.07498
39	N	0.463995	-1.97992	-2.41405	83	C	-6.57837	0.533994	-1.33596
40	C	0.514455	-2.67191	-1.22846	84	C	-6.45716	-1.70425	-0.47562
41	C	1.667558	-2.71917	-0.4054	85	C	-7.93091	0.560615	-0.99129
42	C	-0.68984	-3.30457	-0.7858	86	H	-6.11061	1.400909	-1.79166
43	C	1.497285	-3.39339	0.928235	87	C	-7.79788	-1.70324	-0.12345
44	C	-0.64005	-4.45531	0.184945	88	C	-8.52647	-0.54575	-0.39358
					89	H	-8.51844	1.450234	-1.18814

90	H	-8.27341	-2.55933	0.340361	121	C	-5.83381	2.962986	1.175518
91	H	-9.57885	-0.51244	-0.13144	122	N	-7.42713	4.047202	-0.24789
92	C	4.949351	-0.71803	-2.68854	123	H	-8.18003	3.716726	0.331529
93	H	3.926748	-0.399	-2.48869	124	H	-7.65907	4.697697	-0.97948
94	H	5.398405	0.089499	-3.26775	125	H	-4.29827	2.052074	2.326668
95	C	5.019672	-2.02728	-3.46493	126	H	-6.63769	2.527944	1.759385
96	H	6.064388	-2.22645	-3.72497	127	C	-2.65016	4.62904	-1.19123
97	H	4.700261	-2.85403	-2.82244	128	H	-2.43021	3.882219	-1.96551
98	C	4.158914	-1.9699	-4.71889	129	H	-2.9512	5.540085	-1.71211
99	H	3.101537	-1.83666	-4.4711	130	H	-0.5618	5.15507	-1.00448
100	H	4.454154	-1.13774	-5.36464	131	H	-1.57036	5.737337	0.307006
101	H	4.247133	-2.89059	-5.29967	132	H	-1.31372	-4.23261	1.01974
102	C	-5.84802	-3.98947	0.305809	133	H	-1.04238	-5.3589	-0.28921
103	H	-4.94086	-4.42495	0.725059	134	C	-0.3849	-1.95218	4.553234
104	H	-6.51157	-3.78123	1.148766	135	H	0.705214	-5.27993	1.657457
105	C	-6.51065	-4.94976	-0.67761	136	H	1.336543	-5.32579	0.017331
106	H	-7.42989	-4.49347	-1.05961	137	H	2.466484	-3.57788	1.392203
107	H	-6.81819	-5.83589	-0.11258	138	H	0.929397	-2.7552	1.615078
108	C	-5.60691	-5.34876	-1.8361	139	H	-0.03256	-2.43487	5.46741
109	H	-5.2867	-4.47646	-2.41186	140	C	-1.0943	-2.96472	3.664963
110	H	-4.70832	-5.86073	-1.48058	141	H	-1.06917	-1.16386	4.874548
111	H	-6.12727	-6.02348	-2.52006	142	H	-0.42804	-3.79106	3.407492
112	C	0.873628	-0.59394	-2.61867	143	H	-1.44277	-2.50554	2.737048
113	H	-0.62752	0.317432	-1.36862	144	H	-1.96062	-3.3801	4.185267
114	H	0.848431	-0.15044	-0.51631	145	H	-5.32502	4.998695	-1.51868
115	H	-0.33735	-2.23788	-2.96941	146	H	0.24902	2.372413	-1.66325
116	H	0.387832	-0.25198	-3.53389	147	H	-1.84711	-1.8023	-1.68859
117	H	1.945627	-0.49319	-2.78352	148	H	-3.25211	-4.09708	-0.20604
118	H	-1.52245	-0.16669	3.129902	149	H	2.911733	-1.84714	-1.84261
119	C	-5.09129	4.363474	-0.66965	150	H	3.916375	-2.31992	1.019888
120	C	-6.14444	3.803201	0.081198					

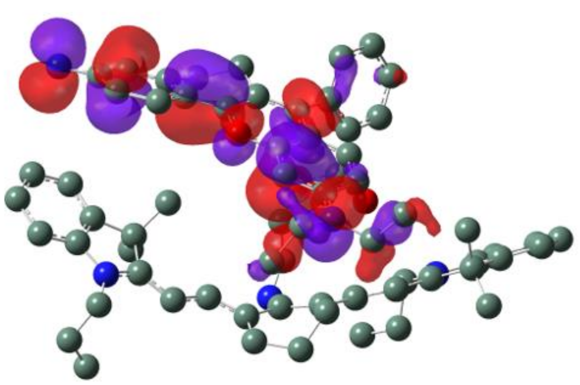

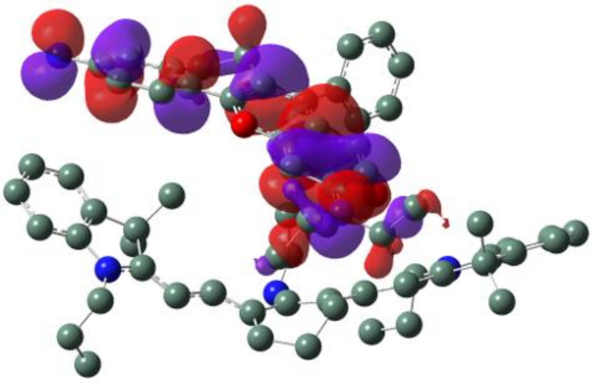

BH^{2+}-263

BH^{2+}-261

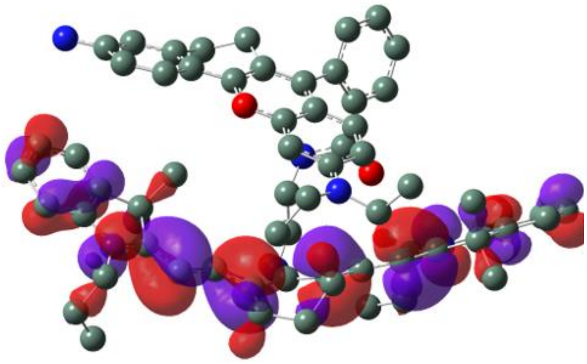

BH^{2+}-265

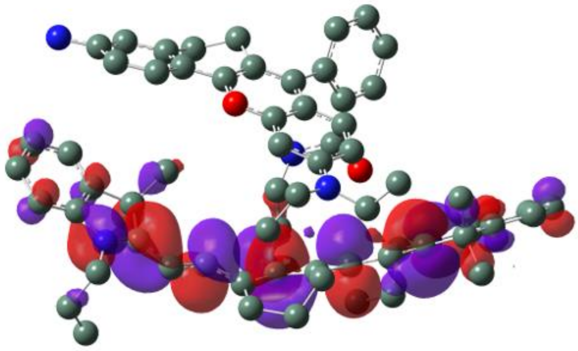

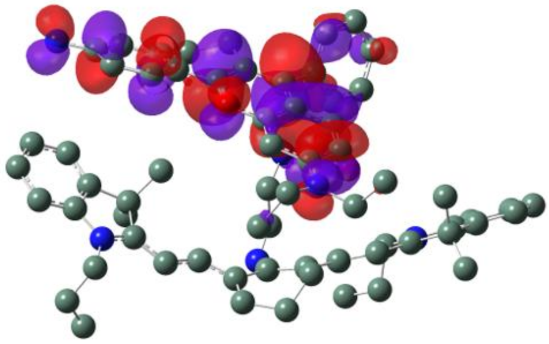

BH^{2+}-266

BH^{2+}-264

Figure S27. LCAO for orbitals 261, 263-266 in probe BH^{2+}.

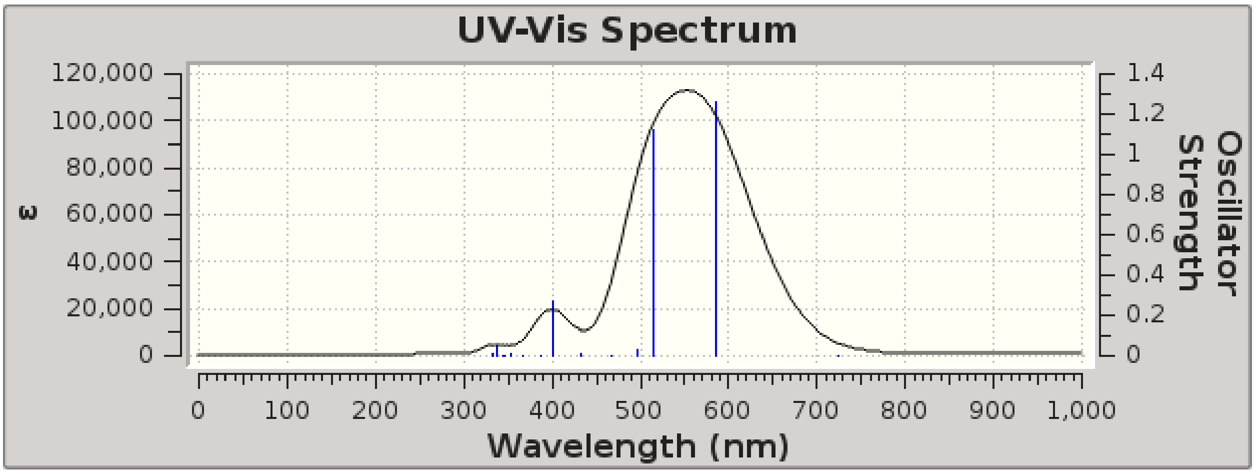

Figure S28. Calculated UV-Vis spectrum for probe BH^{2+}.

Table S8. Excitation Energies and Oscillator Strengths for BH^{2+}.

Excitation energies and oscillator strengths:

Excited State 1: Singlet-A 1.7263 eV 718.22 nm f=0.0008 <S**2>=0.000

264 -> 265 0.70666

This state for optimization and/or second-order correction.

Total Energy, E(TD-HF/TD-DFT) = -3037.89654534

Copying the excited state density for this state as the 1-particle RhoCI density.

```
Excited State   2:      Singlet-A      2.1211 eV   584.52 nm   f=1.2743   <S**2>=0.000
      263 -> 265        0.15505
      264 -> 266        0.68756

Excited State   3:      Singlet-A      2.4148 eV   513.43 nm   f=1.0816   <S**2>=0.000
      263 -> 265        0.68013
      264 -> 266       -0.15396

Excited State   4:      Singlet-A      2.5040 eV   495.14 nm   f=0.0297   <S**2>=0.000
      262 -> 265        0.70208

Excited State   5:      Singlet-A      2.6600 eV   466.10 nm   f=0.0016   <S**2>=0.000
      263 -> 266        0.70393

Excited State   6:      Singlet-A      2.8636 eV   432.96 nm   f=0.0139   <S**2>=0.000
      262 -> 266        0.69030

Excited State   7:      Singlet-A      3.1153 eV   397.98 nm   f=0.2692   <S**2>=0.000
      261 -> 265        0.68420

Excited State   8:      Singlet-A      3.2095 eV   386.31 nm   f=0.0007   <S**2>=0.000
      264 -> 267        0.70419
```

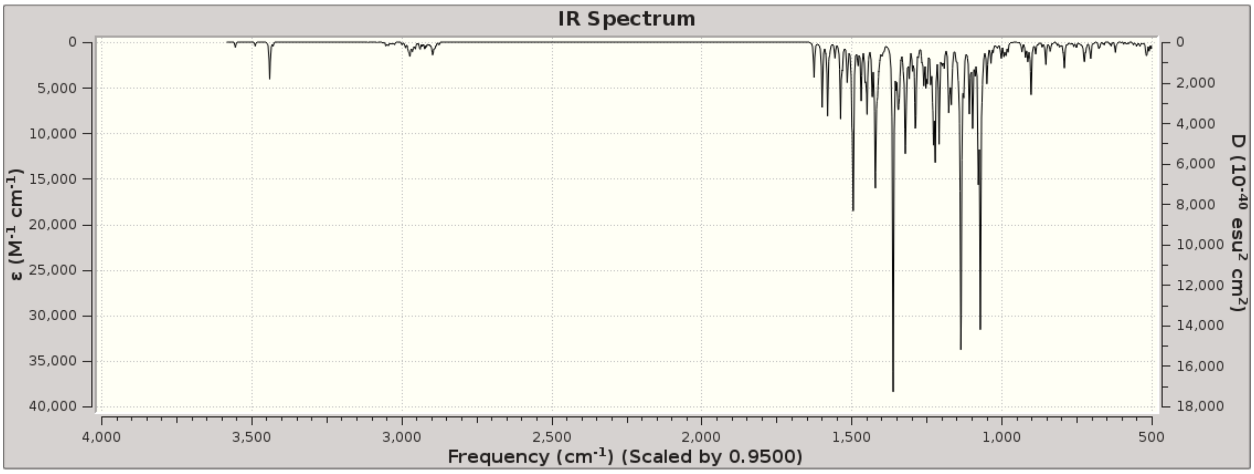

Figure S29. Calculated FTIR spectrum of probe BH^{2+} in water.

Instruments and Materials

Solvents and reagents were obtained from Sigma-Aldrich or Fisher scientific. Column chromatographic purification was conducted on silica gel (200-300 mesh) obtained from Sigma-Aldrich while thin-layer chromatography (TLC) analysis was conducted in silica gel plates obtained from Sigma-Aldrich. Intermediates and the fluorescent probes were characterized by Varian Unity Inova NMR spectrophotometer at 400 MHz and 100 MHz to record 1H NMR and 13C NMR spectra in $CDCl_3$ solution. Absorption spectra were collected by employing Per-kin Elmer Lambda 35 UV/VIS spectrometer while fluorescence spectra were performed on Jobin Yvon Fluoromax-4 spectrofluorometer.

Synthetic route of near-infrared Probes A and B

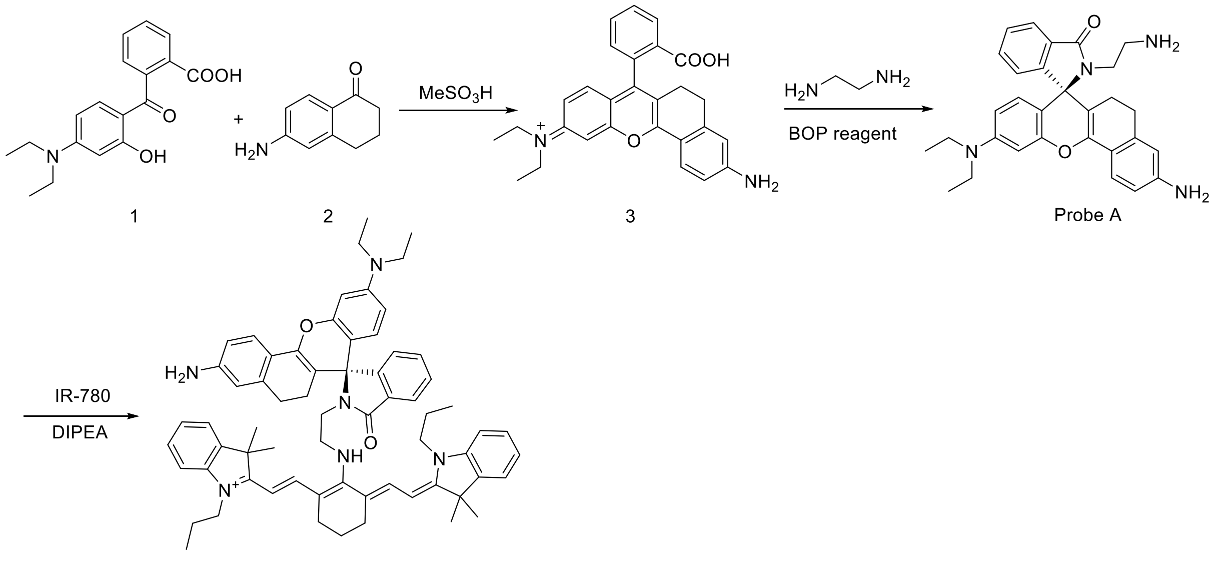

Probe B

Compound 3[2] (439 mg, 1 mmol), Ethylenediamine (180 mg, 3 mmol), BOP reagent (530 mg, 1.2 mmol) and Triethylamine(1 mL) were added to dry DCM (10 ml), the mixture was stirred at room temperature for 16 hours. Then the mixture was diluted with DCM, washed with water and brine, dried with anhydrous Na_2SO_4, filtered and concentrated in vacuo. The resulting residue was purified by using flash column chromatography gradient elution with methanol ratio to dichloromethane from 5% to 10%. The probe A was obtained as blue solid. ^{1}HNMR (300 MHz, $CDCl_3$) δ: 7.78 (d, J = 7.2 Hz, 1H), 7.59 (d, J = 8.2 Hz, 1H), 7.38 (p, J = 7.2 Hz, 2H), 7.09 (d, J = 7.1 Hz, 1H), 6.55 (d, J = 8.2 Hz, 1H), 6.45-6.28 (m, 3H), 6.23 (d, J = 8.7 Hz, 1H), 4.02 (s, 4H), 3.43-3.65 (m, 1H), 3.25-3.30 (m, 5H), 2.78 - 2.56 (m, 2H), 2.46-2.56 (m, 2H), 1.73-1.41 (m, 2H), 1.11 (t, J = 6.9 Hz, 6H); ^{13}CNMR (75 MHz, $CDCl_3$) δ: 169.61, 152.90, 151.57, 148.86, 147.50, 138.31, 132.71, 131.45, 128.55, 128.49, 123.71, 123.63, 123.03, 120.14, 114.21, 112.68, 108.95, 104.77, 100.44, 98.06, 67.20, 44.57, 42.05, 41.18, 28.52, 21.40, 12.93. LCMS (ESI): calculated for $C_{30}H_{32}N_4O_2$ $[M]^+$481.2, found 481.5.

The compound 4 (240 mg, 0.5 mmol), IR780 (400 mg, 0.6 mmol), DIPEA (129 mg, 1 mmol) were added to MeCN (10 ml). The mixture was refluxed for 2 hours, then the mixture was concentrated in vacuo and diluted with DCM, washed by water and brine, dried with anhydrous Na_2SO_4, filtered and concentrated. The resulting residue was purified by using flash column chromatography gradient elution with methanol ratio to dichloromethane from 0% to 5%. The probe B was obtained as blue solid. ^{1}HNMR (300 MHz, $CDCl_3$) δ: 9.28 (s, 2H), 7.83 (d, J = 7.5 Hz, 2H), 7.65-7.47 (m, 5H), 7.18-7.31 (m, 5H), 7.06 (t, J = 7.5 Hz, 2H), 6.87 (d, J = 7.8 Hz, 2H), 6.60 (d, J = 8.2 Hz, 1H), 6.54 (s, 1H), 6.44-6.24 (m, 2H), 5.60 (d, J = 12.7 Hz, 2H), 3.95-4.19 (m, 4H), 3.84-3.69 (m, 4H), 3.65-3.51 (m, 2H), 3.50-3.40 (m, 2H), 3.34 (t, J = 7.4 Hz, 4H), 2.65-2.52 (m, 2H), 2.52-2.39 (m, 4H), 1.84-1.71 (m, 6H), 1.54 (d, J = 10.4 Hz, 10H), 1.14 (t, J = 7.0 Hz, 6H), 0.98 (t, J = 7.4 Hz, 6H); ^{13}CNMR (75 MHz, $CDCl_3$) δ: 170.89, 167.51, 153.27, 149.07, 148.07, 143.25, 140.17, 137.44, 133.54, 129.01, 128.38, 128.24, 124.11, 123.86, 123.01, 122.10, 119.26, 114.15, 112.83, 109.09, 108.97, 98.26, 94.64, 68.13, 53.02, 47.84, 45.14, 44.71, 41.75, 28.92, 28.82, 26.46, 21.85, 21.43, 20.42, 13.00, 12.11. LCMS(ESI):calculated for $C_{66}H_{75}N_6O_2$ $[M]^+$983.5, found 983.5.

^{1}H NMR and ^{13}C NMR spectra of probes A and B

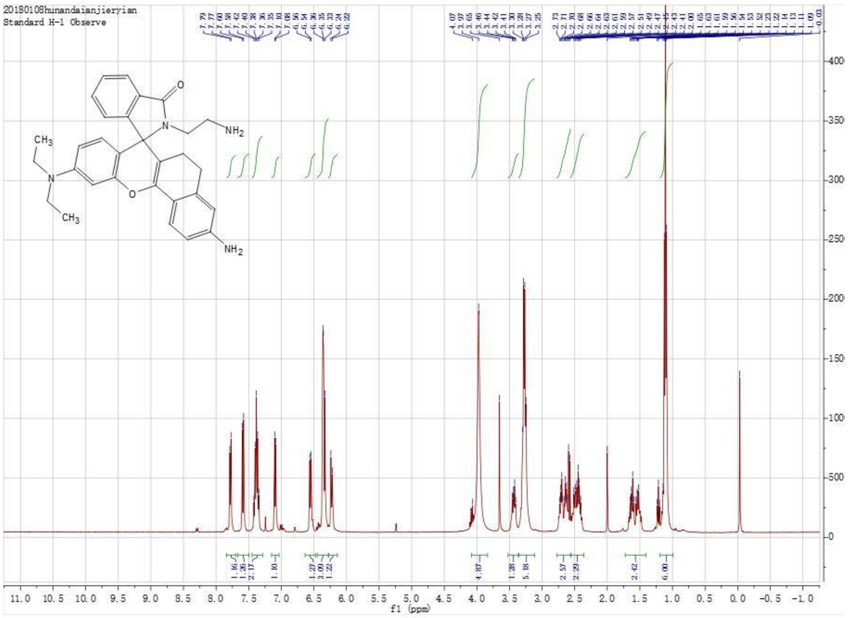

Figure S30. ^{1}H NMR spectrum of probe **A** in $CDCl_3$ solution

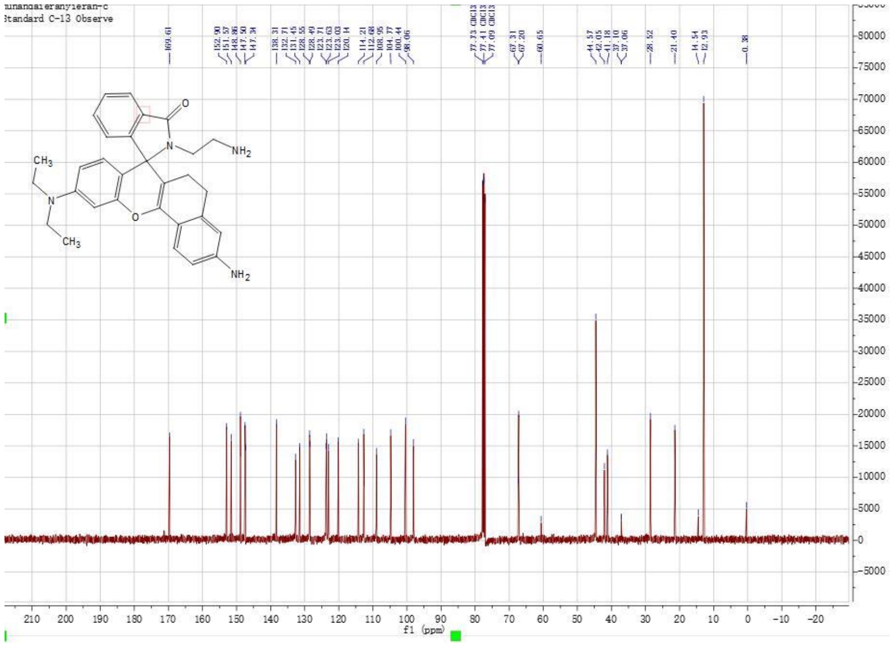

Figure S31. ^{13}C NMR spectrum of Probe A in $CDCl_3$ solution

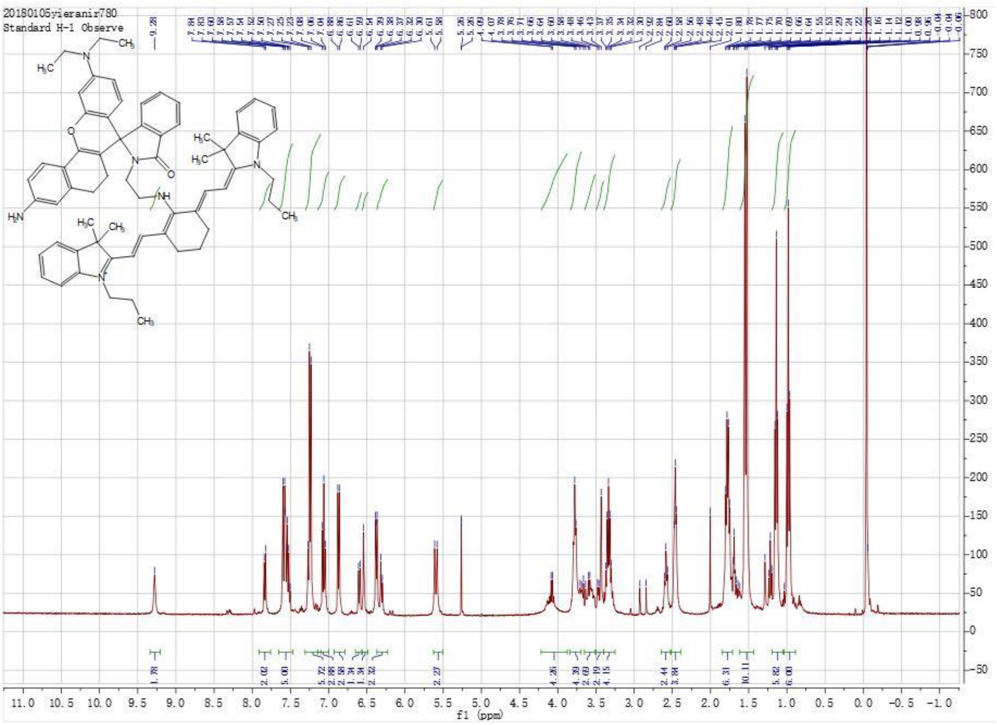

Figure S32. ^{1}H NMR spectrum of Probe B in $CDCl_3$ solution

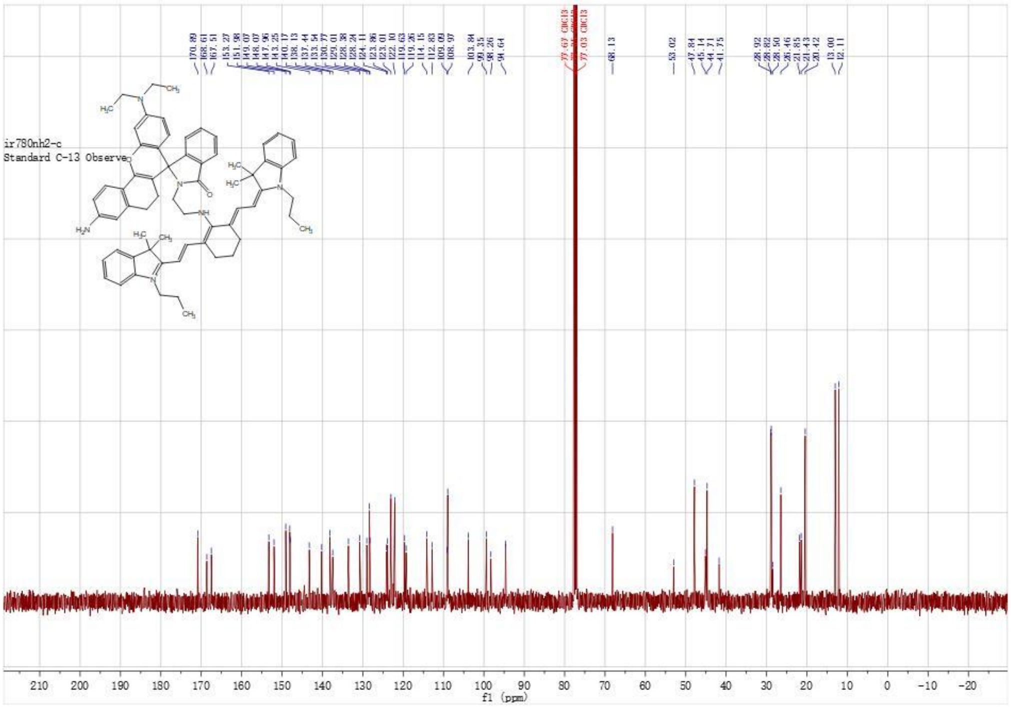

Figure S33. ^{13}C NMR spectrum of probe B in $CDCl_3$ solution

Calculation of fluorescence quantum yields of the probes

We use following equation to determine fluorescence quantum yields of the probes.

$$\phi_x = \phi_{st} \frac{\eta_x^2 \ A_{st} I_x}{\eta_{st}^2 \ A_x I_{st}} \tag{1}$$

ϕ stands for fluorescence quantum yield while I_x represents integration of fluorescence spectra of the probes at specific excitation wavelength. A is the absorbance with optimal ranges from 0.02 to 0.05 under the specific excited wavelength. η is the refractive index of solvents employed for optical measurements, and the subscripts x and st are the probe and a fluorescence reference with known fluorescence quantum yield, respectively.

Rhodamine 6G with fluorescence quantum yield of 95.0% in ethanol was used as standard to calculate quantum yield of probe A. A near-infrared rhodamine dye with a fluorescence quantum yield 22.6% in pH 7.4 PBS buffer with 10% ethanol was used as standard to calculate the quantum yield of probe B[3].

Standard to calculate quantum yield of probe B:

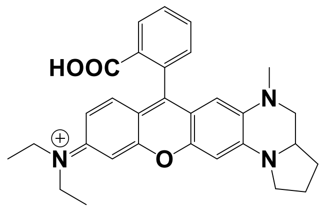

Determination of pKa by fluorometric titration

The constant K_a of probes were obtained by fluorometric titration as a function of pH using the fluorescence spectra. The expression of the steady-state fluorescence intensity *F* as a function of the proton concentration has been extended for the case of a n: 1 complex between H^+ and a fluorescent probe, which is expressed by the equation below:

$$F = \frac{F_{min}[H^+]^n + F_{max}K_a}{K_a + [H^+]^n} \tag{2}$$

F_{min} and F_{max} are the fluorescence intensities at maximal and minimal H^+ concentrations, respectively, and *n* is apparent stoichiometry of H^+ binding to the probe which affects the fluorescent change. Nonlinear fitting of equation expressed above to the fluorescence titration data recoded as a function of H^+ concentration with K_a and n as free adjustable parameters yields the estimated apparent constant of K_a.

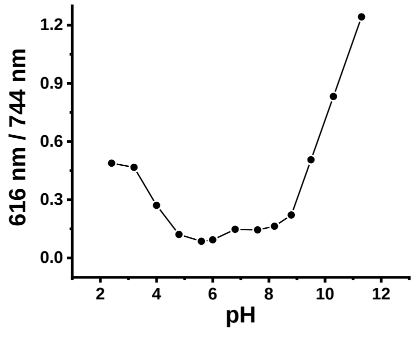

Figure S34. The fluorescence ratio of cyanine acceptor to rhodamine donor of Probe B versus different pH values under rhodamine donor excitation of 450 nm.

Determination of Energy Transfer Efficiency (ETE, %)

The energy transfer efficiency from rhodamine donor to cyanine acceptor was determined by using following equation [4].

$$ETE\% = \frac{\text{Fluorescence quantum yield of the acceptor under the donor excitation}}{Fluorescence\ quantum\ yield\ of\ the\ acceptor\ under\ the\ acceptor\ excitation} \quad (3)$$

References.

1. GaussView; 6, V.; Dennington, R.; Keith, T. A.; Millam, J. M. Semichem Inc. Shawnee Mission, KS, 2016.
2. Ren, T.; Xu, W.; Jin, F.; Cheng, D.; Zhang, L.; Yuan, L.; Zhang, X., Rational Engineering of Bioinspired Anthocyanidin Fluorophores with Excellent Two-Photon Properties for Sensing and Imaging. *Analytical Chemistry* **2017,** *89* (21), 11427-11434.
3. Zhang, Y.; Xia, S.; Fang, M.; Mazi, W.; Zeng, Y.; Johnston, T.; Pap, A.; Luck, R. L.; Liu, H., New near-infrared rhodamine dyes with large Stokes shifts for sensitive sensing of intracellular pH changes and fluctuations. *Chemical Communications* **2018,** *54* (55), 7625-7628.
4. Thivierge, L.; Han, J.; Jenkins, R.M.; and Burgess, K. Fluorescent Proton Sensors Based on Energy Trasfer. The Journal of Organic Chemistry, 2011, 76. 5219-5228.
